# Supplementary figures and images for: Internality and the internalisation of failure: Evidence from a novel task
Source: PLoS Comput Biol. 2021 Jul 6;17(7):e1009134. doi: 10.1371/journal.pcbi.1009134 (PMC8284820; doi:10.1371/journal.pcbi.1009134)

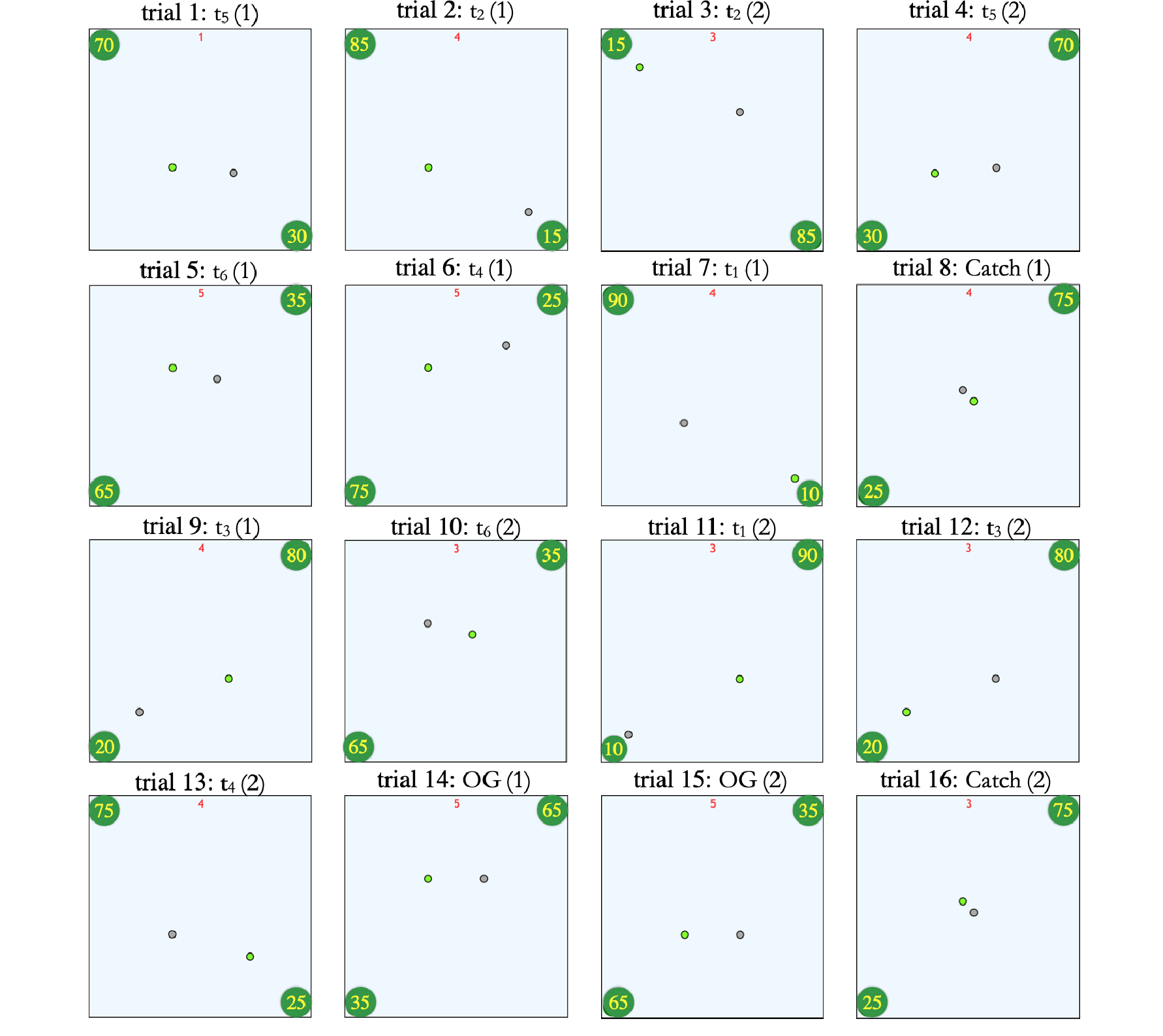

Supplement: S1 Fig — The background of the canvas is light blue, indicating the influence condition (for half of the subjects this would signify high, and for the other half, low, influence). The vehicles and goals for the first 16 trials of a block are displayed as they appear at the start of the trial (vehicle selection phase; the red timer on top denotes time left to choose, random here) with goals made slightly bigger, for illustrative purposes. The ordering of the trials is randomized, as is the position of the goals (the angle by which they are rotated). The labels for each trial type are on top of each screenshot, the number in parentheses being the order of occurrence of this trial type within the block. Note how on the second occurrence (i.e. (2)), the vehicles’ position is swapped. The sixteenth trial (bottom-right) is always a catch trial; but the ordering of all the other trials is random. (TIF) [file pcbi.1009134.s001.tif]

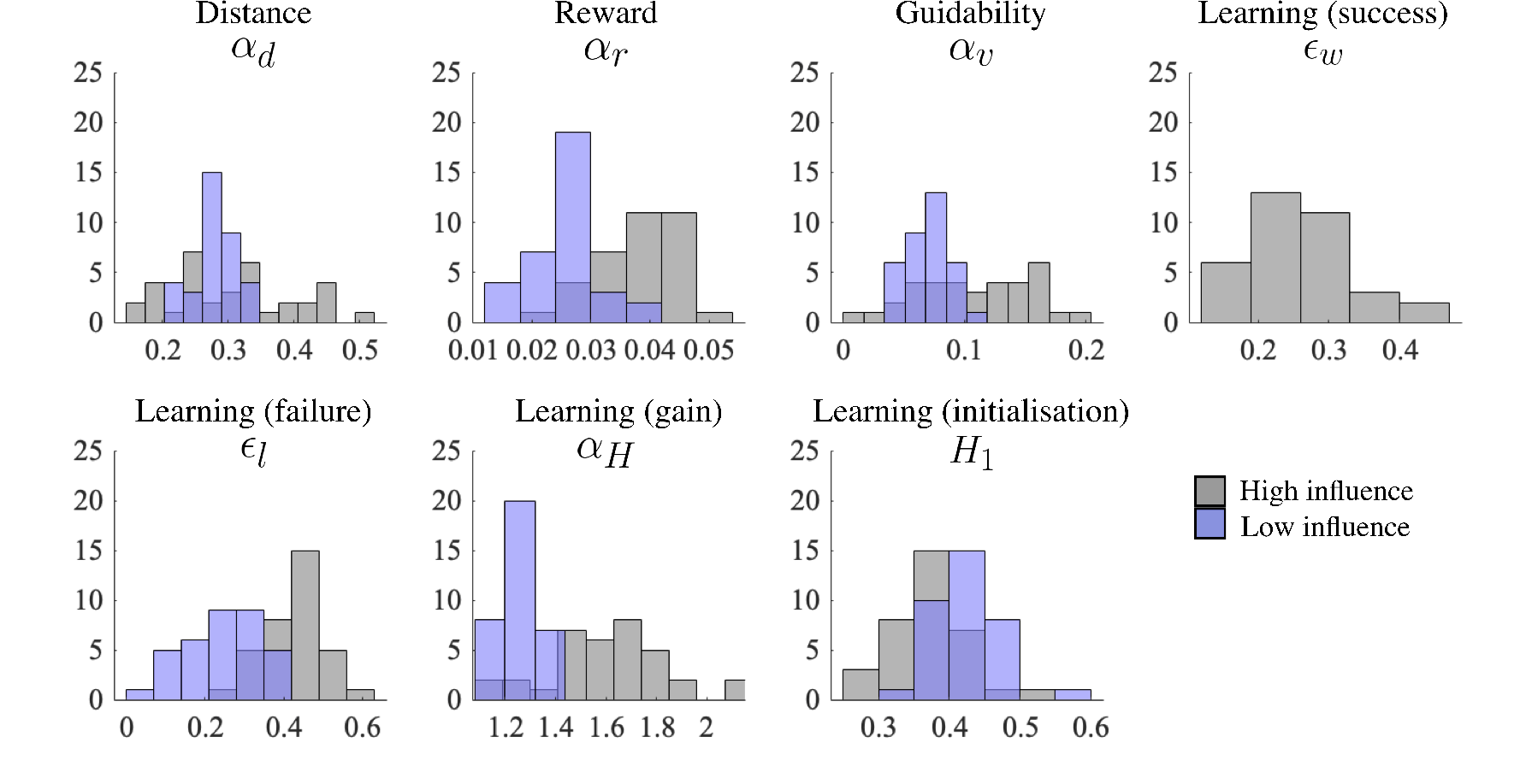

Supplement: S2 Fig — Here, we show the recovered parameter fits for our winning model, obtained via stan. Learning from success (ϵw) is inherently only defined in high influence conditions. All values are on average lower in low influence conditions, possibly due to the greater stochasticity in decision making (this is equivalent to having a lower inverse temperature). H1 values in low influence conditions are slightly higher, possibly compensating for the incapability of the model to describe higher, initial tendencies to choose the riskier goal through other trial-based features. Although learning from failure (ϵl) parameter fits are lower on average in low compared to high influence conditions, recall that this is the only learning feedback we posit for low influence conditions. Thus, the resulting Ht values will, in time, quickly fall below those reached in high influence conditions, as they can only monotonically decrease. (TIF) [file pcbi.1009134.s002.tif]

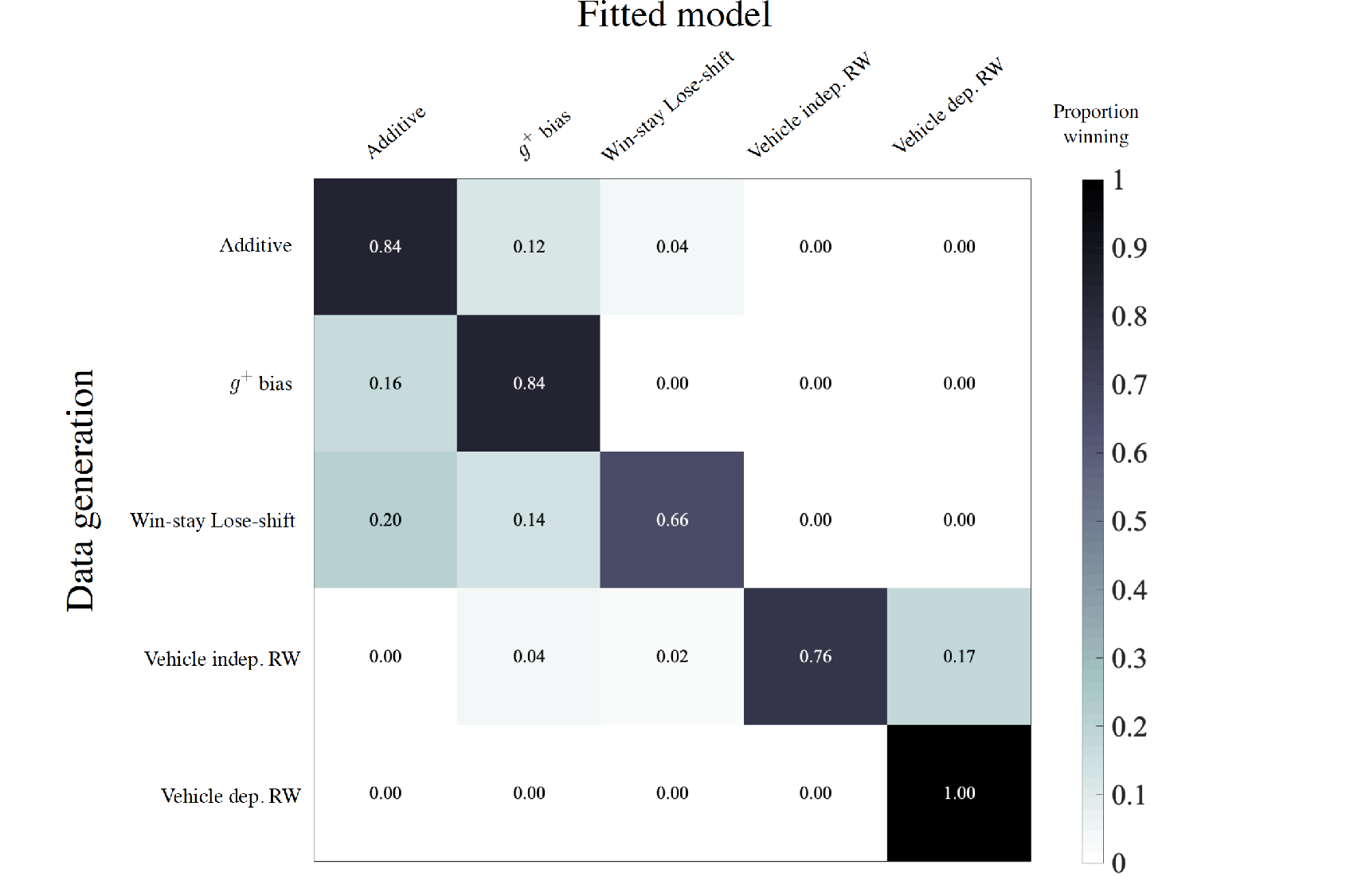

Supplement: S3 Fig — We generated 50 datasets from all our models, and performed model recovery analyses on all models (Palminteri et al. 2017), using WAIC scores. We generated synthetic data from the posterior parameterisation of each model obtained after fitting to the data. Similar models are at higher risk of being confused—however, our winning model (“Vehicle dependent RW”) had an expected probability of 0.04 of being wrongfully recovered if a different formulation were true (false positive rate); and a probability of 1 of being recovered if the data were truly generated by it (true positive rate). (TIF) [file pcbi.1009134.s003.tif]

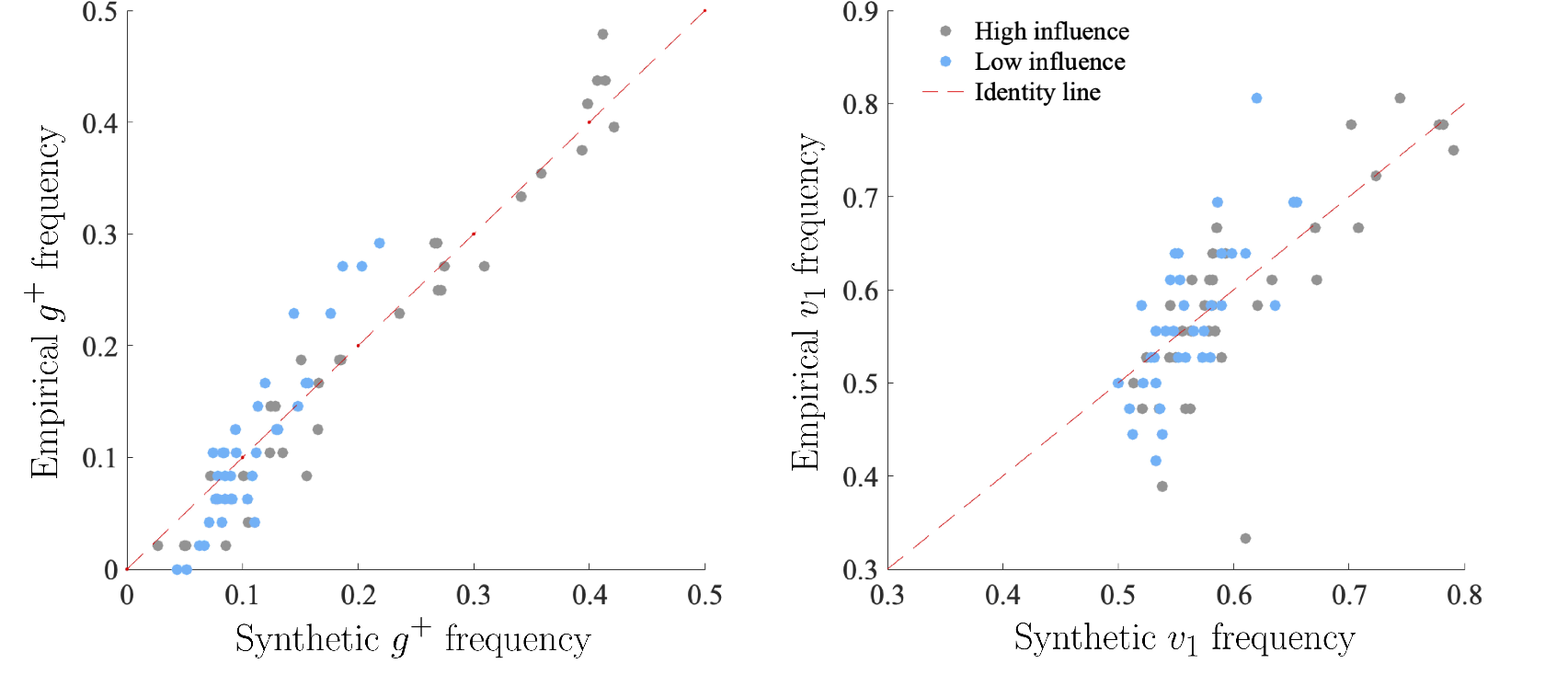

Supplement: S4 Fig — These plots show that the winning model (“Vehicle dep. RW”) generated frequencies of g+ choices (left) and more controllable vehicle v1 (right) are well calibrated to the data. Each dot is a subject’s statistic as computed in a particular influence condition (gray: high; blue: low). The variance explained by the model is high for both measures (g+ and v1 choice frequency) and influence conditions (high and low): (i) g+: high influence, r2 = 0.96, low: r2 = 0.84, (ii) v1: high influence, r2 = 0.69, low: r2 = 0.49. Low influence statistics are less faithfully recapitulated than high influence statistics, possibly on account of subjects inherently attempting to randomise their decision making to obtain more success. Finally, vehicular choice is less faithfully recapitulated in both influence conditions—this is expected, since subjects only have a noisy notion of which vehicle is most controllable. (TIF) [file pcbi.1009134.s004.tif]

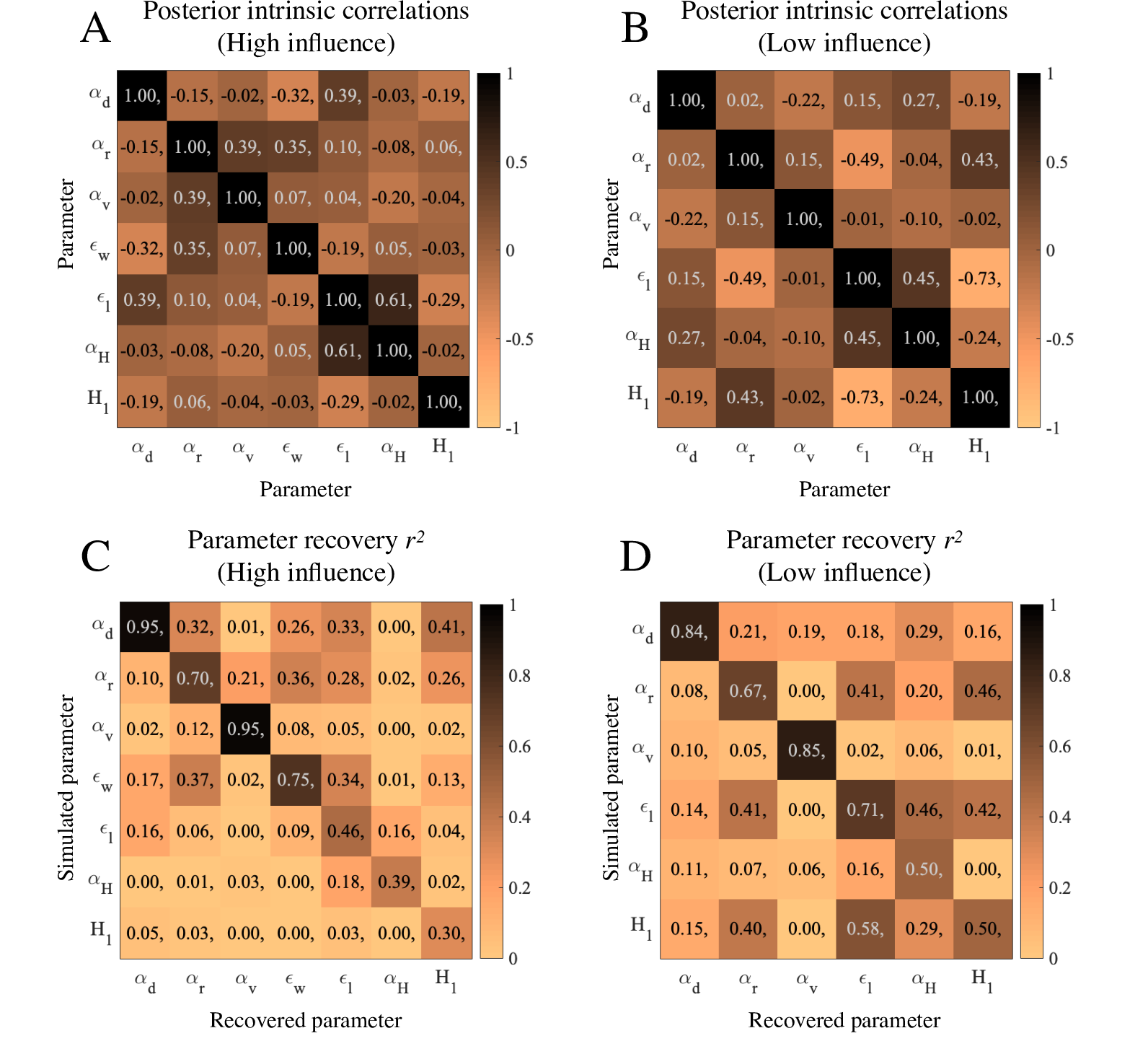

Supplement: S5 Fig — Here, we show recovery analyses results for the winning model parameters (i.e. sensitivities to distance, αd; to reward, αr; to guidability, αv; learning gain, αH; learning rates from success, ϵw; from failure, ϵl; and the initialisation, H1). The confusion matrices exemplify the intrinsic correlations in the posterior parameter means (A; high influence and B; low influence conditions) and the capacity of our fitting procedure to to recover the original parameters when these generate synthetic data (150 datasets; C, high influence; D, low influence conditions). For the latter two insets, we chose to report the variance explained or r2 as a clearer measure of the amount of information about the original parameters that is carried by the recovered parameters. Parameters ϵl and αH exhibit rather large correlations in the posterior (inset A). However, recovery is good in high influence conditions, as the variance explained for all parameters is maximal for the corresponding parameter (inset C). In particular with respect to our results and conclusions, recovery of the ϵl parameter is sufficiently good in high influence conditions. In low influence conditions, we find a measure of confusion between parameters H1 and ϵl, which are respectively the initialisation, and the learning (from failure) components of the Rescorla-Wagner component of the model. Thus, functional interpretations of individual differences here should be interpreted with caution. (TIF) [file pcbi.1009134.s005.tif]
