# Supplementary material for: Internality and the internalisation of failure: Evidence from a novel task
Source: PLoS Comput Biol. 2021 Jul 6;17(7):e1009134. doi: 10.1371/journal.pcbi.1009134 (PMC8284820; doi:10.1371/journal.pcbi.1009134)
Supplement: S1 Text — (PDF) [file pcbi.1009134.s006.pdf]

S1 Text

Model-dependent analyses: double-goal trials. We first posited that subjects simply choose according to their preferences for achievability and reward. Thus, our basic modelling approach combined, additively, the main trial features driving choice, i.e. distance, guidability and reward. We then incrementally (using model complexity control) built more sophisticated, and better performing, models, to uncover temporal dependencies across trials (learning).

In its most basic form, $U_t(v, g)$, the utility of choosing vehicle v to attain goal g at trial t, is modelled as follows:

$$U_t(v, g) = -\alpha_d \cdot d_t(v, g) + \alpha_v \cdot 100\gamma_t(v) + \alpha_r \cdot r_t(g) \tag{1}$$

The expression considers the values, at trial t, of the euclidean distance between vehicle v and goal g (i.e. $d_t(v, g)$; which is obtained by dividing the Manhattan distance by 2 and multiplying by $\sqrt{2}$), the guidability of v as inferred by trial t (i.e. $\gamma_t(v)$; this is multiplied by 100, so that the guidability values lie in the same range as distances and rewards, and parameter estimates are comparable), and of the reward connected to g, i.e. $r_t(g)$. There are, of course, four possible choices of vehicle-goal pairs in all DGTs. A subject could in fact choose either vehicle (v_1 or v_2) and aim for either goal (g^- or g^+). The probability of each option is modelled as a generalization of the softmax function for four options (an ordinary multinomial logistic regression):

$$P(v, g) = \frac{e^{U_t(v,g)}}{\sum_{v' \in \{v_1, v_2\}, g' \in \{g^-, g^+\}} e^{U_t(v',g')}} \tag{2}$$

This is our additive model for DGTs ("Additive"), and constitutes the starting structure of *all* our models. In turn, these differ according to the ways they refine $U_t(v, g)$, adding extra features.

In the task, distances and rewards are directly observed. However, vehicle guidability (i.e. $\gamma_t(v)$) has to be learned. We consider a Bayesian form of learning for both vehicles, as follows:

$$\gamma_t(v) = \frac{4}{3}R - \frac{1}{3}$$
$$\text{where } R = \frac{\sum_{\tau=1}^{t-1} n_{good}(\tau)}{\sum_{\tau=1}^{t-1} n_{good}(\tau) + \sum_{\tau=1}^{t-1} n_{bad}(\tau)} \tag{3}$$

$n_{\{good,bad\}}(t)$ gives the number of {good,bad} moves at trial t. R is the perceived probability that the vehicle will go in exactly the direction we press towards. Note that this can arise both as a consequence of the vehicle actually following our command, i.e. $\gamma_t(v)$, or because the vehicle *randomly* went in the direction we asked it to, i.e. $\frac{1}{4}(1 - \gamma_t(v))$. So to infer $\gamma_t(v)$ from the statistic R we need to solve $R = \gamma_t(v) + \frac{1}{4}(1 - \gamma_t(v))$, which yields the first line of the equation. At trial 1, when either vehicle is fully unknown, we set $n_{good} = 1$ and $n_{bad} = 1$, yielding a controllability $\gamma_1(v) = 0.33$. We also explored the possibility of drawing these initial values from top level priors, but ultimately kept them fixed, for simplicity.

In sum, our basic model simply treats additively the set of factors driving choice. At first glance, one might argue that since distance and reward are anti-correlated by design, inference will face an unidentifiability issue, as sensitivities to distance and reward are confounded (increasing sensitivity

to reward or diminishing that to distance would yield approximately the same log-likelihood); however, trial type OG (see e.g. figure S1), which appears twice in DGTs, was conceived to resolve exactly this issue, as it makes the larger goal closer to both vehicles, thereby breaking the anti-correlation.

Learning of achievability in DGTs

We added extra model components to account for learning of achievability throughout the task, reasoning that we might well observe aspects of model-free learning, on top of value-based choice. We then decided to keep the additive structure of equation (17) and add a time-dependent component.

Winning model ("Vehicle dependent RW"). In DGTs, in all but 'Catch' trials, one vehicle (v_{c_t}) is at the center of the canvas, while the other is closer to the less rewarding goal (g^-). We then hypothesized that the main form of learning would be about the success in achieving g^+ using the central vehicle, as subjects are faced with the recurrent question whether g^+ will be achievable with v_{c_t} (empirically, most attempts to g^+ took place utilising the central vehicle, i.e. 92%). Recall that the distance (36 Manhattan steps, or $18\sqrt{2}$ euclidean) between the central vehicle and g^+ was always the same, so the main learning factor underlying choice of g^+ could only be connected to the the vehicle itself. This form of achievability is essentially model-free, and only depends on the experience gathered with the central vehicle.

To include a learning term, and test whether its use is justified, we kept all the "static" aspects of decision making as per the basic structure in equation 17, simply adding an extra component $H_t(v_{c_t})$ to the expression for $U_t(v_{c_t}, g^+)$, leaving unchanged the remaining three terms:

$$U_t(v_{c_t}, g^+) = -\alpha_d \cdot d_t(v_{c_t}, g^+) + \alpha_v \cdot \gamma_t(v_{c_t}) + \alpha_r \cdot r_t(g^+) + \alpha_H H_t(v_{c_t}) \tag{4}$$

where $H_t(v_{c_t}) \in [-1, +1]$ is the output of a learning rule (see below) that integrates the history of successes and failure, and α_H is a parameter ("learning gain") that quantifies the effect of this term. We should also point out that $d_t(v_{c_t}, g^+)$ is simply equal to $18\sqrt{2}$, a constant, since the distance between the central vehicle and g^+ is fixed throughout.

Of course, many learning processes could be suitable, so there is a question as to how H_t should be updated given success and failure. The simplest possible learning rule is the Rescorla-Wagner (RW; delta rule) update, according to which subjects start out on the initial trial of the task with an initial guess for the achievability of g^+ from the center, $H_t(v_{c_t})$ (i.e. H_1). This would then evolve on a trial-by-trial basis. On each trial, there are 8 scenarios that this learning rule should consider: a subject could choose either vehicle, aim for either goal, and face either of two fates.

In the winning model, the notion of success and failure which affect $H_t(v_c)$ is as follows. We regard, as success, only that in gaining the g^+ goal (regardless of whether the central or the other vehicle was chosen), and as failure, any failure both in an attempt to a g^- or g^+ goal. This is because g^- is invariably easier to reach than g^+, so a loss when attempting the easier goal should rationally imply that g^+ is less achievable than we thought. Conversely, succeeding in attaining g^- does not imply that g^+ will be more achievable. Note that since we only count successes on g^+ goals towards the update of $H_t(v)$, learning from wins is not meaningful in low influence blocks, where all (but three attempts across all datasets in total) to g^+ led to a loss. Thus, as one would expect, learning of the hopeless achievability of g^+ in low influence conditions only happens through failures. We have of course tried variations of this model which took into account different learning

speeds for wins and losses faced when attempting g^- and g^+. While we do not report all of these here, for brevity, their performance was not as competent as our final formulation.

Thus, in the winning model, the evolution of $H_t(v)$ across trials is:

$$H_t(v) = H_{t-1}(v) + \begin{cases} 0 & \text{if achieved } g^- \text{ using } v \text{ at } t-1 \\ \epsilon_w \left(+1 - H_{t-1}(v)\right) & \text{if achieved } g^+ \text{ using } v \text{ at } t-1 \\ \epsilon_l \left(-1 - H_{t-1}(v)\right) & \text{if lost using } v \text{ at } t-1 \end{cases} \tag{5}$$

where $t \in \{1 \dots 16\}$ indicates the current trial, and v identifies the vehicle that was chosen at trial $t-1$. For the vehicle that was *not* chosen at trial $t-1$ the value for H_t is simply copied over, i.e. if we denote this vehicle as $\bar{v}$, then we have $H_t(\bar{v}) = H_{t-1}(\bar{v})$. ϵ_w (ϵ_l) are the learning rates for success (failure). This expression keeps track of a surrogate value for the probability of achieving g^+. As such, it does not distinguish between the amount won or loss in the previous trial.

As we model learning throughout the task, at the end of each block in high/low influence conditions, the achievability value for each of the two vehicles involved, i.e. $H_t(v)$ is averaged and used as the starting value for the next block of the same influence conditions.

Other models. Prior to testing our final formulation, we tested several simpler learning schemes. Aside, of course, from our basic additive model (equation 17) we tested four other ways to refine this, which we describe below. These models' out of sample performances as measured by average leave-one-subject-out likelihoods are reported in the main text.

- *Bias.* The bias model is simply the basic structure of equation 17, endowed with a free parameter to account for a propensity to choose g^+ that is not dependent on any other characteristic of the trials. Thus, it simply adds a term to only the utility of attaining g^+ with the central vehicle:

$$U_t(v_{c_t}, g^+) = \alpha_d \cdot d_t(v_{c_t}, g) + \alpha_v \cdot \gamma_t(v_{c_t}) + \alpha_r \cdot r_t(g) + \beta_{bias} \tag{6}$$

  The term in question is of course β_{bias}.

- *WSLS.* The Win-stay Lose-shift formulation only has a 1-trial memory to (de)incentivate choice of g^+ with a vehicle. In this case $H_t(v_{c_t})$ evolves as:

$$H_t(v_{c_t}) = \begin{cases} \epsilon_w & \text{if achieved } g^+ \text{ using } v_{c_t} \text{ at } t-1 \\ \epsilon_l & \text{if lost using } v_{c_t} \text{ at } t-1 \end{cases} \tag{7}$$

  Of course, $\epsilon_w > 0$ and $\epsilon_l < 0$.

- *Vehicle-independent RW.* The last alterantive, which is perhaps closest to the winning model is a Rescorla-Wagner rule that does *not* keep separate records for the two vehicles available inside a block. Here, achieving g^+ with a vehicle will simply increase the value for choosing g^+ regardless of which vehicle is used. That this model performs worse than our winning model means that subjects must be using the different vehicles' histories of achievements to make their decisions, and are not simply being motivated by success and failure to try again (or

quit trying) to achieve g^+. Here, the H_t term drops the dependency on vehicles, so equation 5 becomes:

$$H_t = H_{t-1} + \begin{cases} \epsilon_w \left(+1 - H_{t-1}\right) & \text{if achieved } g^+ \text{ at } t-1 \\ \epsilon_l \left(-1 - H_{t-1}\right) & \text{if lost at } t-1 \end{cases} \tag{8}$$

Model-dependent analyses: single-goal trials. In SGTs, the expression for U (i.e. equation 17) omits the term concerning reward, since only one goal is available. Further, because of the large number of preceeding DGTs, we make the assumption that $\gamma_t(v)$ has fully converged to the true controllability value of the vehicle. Furthermore, to establish whether in each block the H_t term from DGTs would skew decision making during the subsequent single-goal phase, we endowed all SGT models with an ulterior term, which reflects the history of achievement of each vehicle as recorded on the last double-goal trial of the block - i.e. $H_{16}(v)$. This term was found to improve model performance, and was therefore added to all modelling instantiations. The basic additive model for SGTs is then:

$$U(v) = \alpha_d d_t(v) + \alpha_v 100\gamma(v) + \alpha_H H_{16}(v) \tag{9}$$

here $\gamma(v)$ is the true value for the controllability of the vehicle, that is, one of 0.28,0.36,0.43.

Our interaction model (the winning model for SGTs) posits of course an interaction term:

$$U_t(v) = -\alpha_d d_t(v) + \alpha_v 100\gamma(v) + \alpha_{int}\gamma(v)d_t(v) + \alpha_H H_{16}(v) \tag{10}$$

$\gamma(v)d_t(v)$ is an interaction term of guidability and distance.

Finally, we have the probability (P) model, which only used sheer probability as a predictor. Here, the expression for U is simply $U(v) = \alpha_p\omega(\gamma(v); d_t(v); f) + \alpha_H H_{16}(v)$. In this expression, ω denotes the probability of reaching the goal when choosing vehicle v and pressing in an optimal manner. This term, whose approximation we also describe in SMs, depends on the distance from the goal (d), the controllability of the vehicle $\gamma(v)$, and the frequency of pressing that the subject expects to exert over the vehicle. We allowed for subjects' frequencies to vary, so that the f that ω depends on is indeed a free parameter which depends on subject and condition. In high (low) influence conditions individual f parameters were drawn from fixed, top-level normal distributions with mean 8 (4) and standard deviations of 0.1.
