# Supplementary material for: Internality and the internalisation of failure: Evidence from a novel task
Source: PLoS Comput Biol. 2021 Jul 6;17(7):e1009134. doi: 10.1371/journal.pcbi.1009134 (PMC8284820; doi:10.1371/journal.pcbi.1009134)
Supplement: S2 Text — (PDF) [file pcbi.1009134.s007.pdf]

S2 Text

Computation of probabilities. In our task, the probability of reaching a goal with a certain vehicle can be computed in two ways. In our model agnostic analyses, and when fine tuning the task (e.g. to choose the guidability levels of the vehicles), we used synthetically derived proportions of successful attempts. We obtain these by simulating episodes in which artificial subjects press at a uniform frequency throughout the trial ($8Hz$ for high, $4Hz$ for low, influence conditions), taking into account the specific guidability of the vehicle and the distance separating it from the goal. We then simply count the number of times (and divide by the number of simulated episodes, i.e. 1000) that the vehicle made it to the goal in time. In our model-based analyses of SGTs, however, we wanted to take into account possible, subtle variations in the way subjects might have perceived their pressing frequency to be higher or lower than that allowed. This "subjective prospective frequency" is then a free parameter, which we allow to vary across subjects. While models using pure probability did not account for the data most competently (and where thus discarded for DGTs analyses), for completeness (and replicability), we report our procedure to find a model-based closed form for the probability, which a sampling algorithm such as that implemented in stan can use to compute probabilities on the fly.

Thus, in SGTs, models using probability of success as a predictor generate decisions based on the estimated probability of achieving the goal. Let us call this probability $\omega(\gamma(v); d; f)$, where $\gamma(v) \in \{0.28, 0.36, 0.43\}$ is vehicle v's guidability, that is, the probability that it will move in the intended direction vs. uniformly at random (we assume this value to be fully known by the time SGTs are faced); d is the distance between the vehicle and its goal, and f is the prospective frequency individual to each subject and influence condition.

In order to be able to make inferences about f in our hierarchical Bayesian model, we built a closed form expression or approximation for the way that pressing frequency determines these probabilities (assuming optimal choices). We obtained this using the following procedure:

1. We simulated synthetic trials in which an artificial subject made optimal pressing choices at each frequency in the range $[1 : 0.1 : 10]$ (1 to 10 in steps of $0.1Hz$), with vehicle-goal distances in the range $[4 : 1 : 36]$ Manhattan steps, and vehicle controllabilities in the range $[0.1 : 0.025 : 0.5]$.

2. We computed the empirical probabilities for each combination of these parameters to reach the goal by running 5000 simulated trials from each.

3. Now that we have the empirical correspondences between distance, guidability, frequency of pressing, and probability of success, we can build a simple model to predict this latter as precisely as possible. We then (over)fitted a third degree multivariable polynomial (the outcome of which then goes through a logit function), to the empirical outcome probabilities; this included all three variables of vehicular controllability, distance and frequency as covariates. Thus, if for brevity we call $\gamma := \gamma(v)$, and $f := f_{s;c}$,we have $K(\gamma; d; f)$ as our polynomial:

$$K(\gamma; d; f) = a_0 + a_1\gamma + a_2 d + a_3 f + a_4\gamma d + a_5\gamma f + a_6 df \cdots + a_{16}\gamma^3 + a_{17}d^3 + a_{18}f^3 \quad (11)$$

   The polynomial includes 19 terms in total. The probability is then computed as: $\omega(\gamma; d; f) = \sigma\left(K(\gamma; d; f)\right)$ where σ is the logistic sigmoid function.
