# Supplementary material for: Internality and the internalisation of failure: Evidence from a novel task
Source: PLoS Comput Biol. 2021 Jul 6;17(7):e1009134. doi: 10.1371/journal.pcbi.1009134 (PMC8284820; doi:10.1371/journal.pcbi.1009134)
Supplement: S3 Text — (PDF) [file pcbi.1009134.s008.pdf]

S3 Text

Feature-based learning models To determine whether participants impute outcomes to individual trial features, rather than vehicle value (i.e. the term H_t in our winning model), we evaluated an alternative class of models in which subjects' sensitivities to individual trial features (i.e. distance, guidability and reward) evolve over time in a reward-driven manner. The sensitivies are of course those to (1) distance (model m_d), (2) reward (model m_r) and (3) guidability (model m_v). These models posit that (1) failures might sensitise (and successes, de-sensitise) participants to distance, or (2) failures might de-sensitise (successes, sensitise) participants to rewards. We did not hold particular expectations with respect to how guidability might evolve in a reward-dependent manner, because it is unclear whether wins/losses should sensitise or de-sensitise participants to guidability (i.e. guidability is a feature both functional to achieving goals, and avoiding failures).

To implement these models we utilised a very similar update scheme to the one we introduced for the H_t term, with the crucial difference that achievement and failure affect the sensitivity to trial features rather than a separate achievability component. For these models, choice of the vehicle v and goal g at trial t arises as a softmax policy which takes as input the term $U_t(v, g)$ defined below. For model m_d:

$$U_t(v, g) = -\alpha_d(t) \cdot d_t(v, g) + \alpha_v \cdot 100\gamma_t(v) + \alpha_r \cdot r_t(g) \tag{12}$$

in which the sensitivity to distance (i.e. $\alpha_d(t)$) evolves in a way that depends on reward, and the other α's, weighing guidability (α_v), and reward (α_r) are fixed. Note that we enforce that $\alpha_d(t)$ never falls below zero in our model code.

$$\alpha_d(t) = \alpha_d(t-1) + \begin{cases} 0 & \text{if achieved } g^- \\ -\omega_w & \text{if achieved } g^+ \\ +\omega_l & \text{if lost} \end{cases} \tag{13}$$

Model m_r:

$$U_t(v, g) = -\alpha_d \cdot d_t(v, g) + \alpha_v \cdot 100\gamma_t(v) + \alpha_r(t) \cdot r_t(g) \tag{14}$$

in which the sensitivity to reward evolves in a way that depends on reward.

$$\alpha_r(t) = \alpha_r(t-1) + \begin{cases} 0 & \text{if achieved } g^- \\ +\omega_w & \text{if achieved } g^+ \\ -\omega_l & \text{if lost} \end{cases} \tag{15}$$

In this formulation losses desensitise, while wins sensitise, participants to rewards.
Finally, model m_{veh}:

$$U_t(v, g) = -\alpha_d \cdot d_t(v, g) + \alpha_v(t) \cdot 100\gamma_t(v) + \alpha_r \cdot r_t(g) \tag{16}$$

in which the sensitivity to guidability evolves in a way that depends on reward.

$$\alpha_v(t) = \alpha_v(t-1) + \begin{cases} 0 & \text{if achieved } g^- \\ +\omega_w & \text{if achieved } g^+ \\ +\omega_l & \text{if lost} \end{cases} \tag{17}$$

Note that here ω_w and ω_l are allowed to be positive or negative, whereas in the other two formulations (i.e. m_d and m_r) they are forced to be positive or equal to zero. These models posit the learning of a single feature. To establish whether subjects might have been learning the value of multiple features at once, we simply combined the learning schemes above. There were then three models in which subjects learned about 2 features (m_{d+r}, combining distance and reward value learning; m_{d+v}, combining distance and guidability value learning; and finally m_{r+v} combining guidability and reward value learning). One last model learned about all features (i.e. m_{d+r+v}).

All models performed worse than our winning model according to WAIC. The table below holds a summary of the WAIC scores obtained.

**Model**	**WAIC score**
Vehicle dep. RW	5118
m_d	5135
m_r	5140
m_v	6391
m_{d+r}	5214
m_{d+v}	5130
m_{r+v}	5223
m_{d+r+v}	5216

Table 1: Model comparison for models with outcome-dependent evolution of distance (i.e. m_d), guidability (m_v), reward sensitivities (i.e. m_r), and their combinations.
