# Supplementary material for: Internality and the internalisation of failure: Evidence from a novel task
Source: PLoS Comput Biol. 2021 Jul 6;17(7):e1009134. doi: 10.1371/journal.pcbi.1009134 (PMC8284820; doi:10.1371/journal.pcbi.1009134)
Supplement: Attachment — Submitted filename: ResponseToReviewers.pdf [file pcbi.1009134.s010.pdf]

Response to reviewers

Dear reviewers,

We thank you very much for your efforts and insight in reviewing our manuscript, entitled 'Subjective Beliefs In, Out, and About Control: A Quantitative Analysis'. Please find our response below, with original comments by the author in cursive, our response to each comment in normal font, and changes to the manuscript in bold font. When contents of tables, figures, or equations, are changed, we will explicitly provide a reference to the corresponding element in text. All changes have been added to the document using red font, so that these are identifiable.

Reviewer 1

We appreciate being part of this pilot trial. We have provided all material relevant to the replication of our simulations in the folder `reproducibility_pilot_materials`.

Reviewer 2

The authors develop a novel task to measure how people learn action-outcome controllability when making goal-directed decisions, and how these judgements are related to beliefs about locus of control (LoC). They found that participants incorporated a model-free estimate of the value of different agents (cars they would drive towards different goals), and that it was the learning of these agents that was most strongly associated with 'internalizing' factors of LoC. A major finding was that high LoC allowed subjects to quickly learn when a task was unachievable, so that they did not incur further loss and affording an attribution of failure to external rather than internal causes.
Overall, it's an interesting task, analysis, and interpretation but there are some substantial concerns that need to be addressed.

We are grateful for the kind words, and have attempted to address all the substantial concerns with new analyses and text.

Point 1. *More could be done to ensure that the RW components reflect the learning of vehicle value, and not learning the properties of each vehicle. This concern arises because the reward information that's used to teach the vehicle value depends in part on the vehicle properties (esp. guidability). When people get a bad outcome, how do we know that they blame the vehicle as a whole, and not blame a property of the vehicle. A deeper investigation into this learning process may help inform the surprising relationship between internalizing and learning.*

To address this point, we performed additional analyses and introduced additional models. We note that in order to perform a timely model recovery with a satisfactory number of simulations, we employed the Widely Applicable Information Criterion (WAIC) rather than leave-one-subject-out (LOSO) for model selection. Thus, we removed individual trial log-likelihood model comparisons from the Supplementary Information, relying solely on WAIC. However, since readers may find the comparison of the two methods interesting, we still report LOSO results along with WAIC scores. The two measures largely agree, with the only disagreement over models "Vehicle independent RW" and "Win-stay Lose-shift" (see table 1). We edited the methods section to inform the reader that we are using WAIC scores:

> [. . .]**Model assessment was performed via the Widely Applicable Information Criterion (Watanabe & Opper, 2010), and for completeness, also via leave-one-subject-out cross validation (LOSO), i.e. obtaining average log-likelihoods for each subject as they were held out of training and only used as test data. WAIC scores were used to perform model-recovery analyses on all our models, the results of which are reported in Figure S3 (Palminteri et al., 2017)** [. . .]

We modified table 2 and caption (i.e. "Model comparison for DGTs") in text, adding WAIC scores for all models of DGTs. Thus, table 2 in text then becomes table 1 below.

Models	LOSO	WAIC
Additive (1)	0.623	5213
g^+ bias (2)	0.632	5180
Win-stay Lose-shift (3)	0.644	5179
Vehicle independent RW (4)	0.637	5143
Vehicle dependent RW (5)	0.653	5118

Table 1: **Model comparison for DGTs. We report out of sample subject-wise likelihoods (LOSO scores) and WAIC scores for all our models (lower scores are better). Starting from the easiest (additive), which forms the basis for all our models, to the most complex (and best performing). LOSO and WAIC only disagree over models 3 and 4, with WAIC preferring number 4.**

(a) The authors should determine whether participants use reward feedback to update their weights on different properties (influence, guidability, distance), in order to better distinguish where participants assign credit for bad performance.

To determine whether participants impute outcomes to individual trial features, rather than vehicle value, we evaluated three alternative models, in which (1) distance (model m_{dist}), (2) reward (model m_{rew}) and (3) guidability sensitivities (model m_{veh}) evolve over time, in a reward-dependent manner. These models posit that (1) failures might sensitise (and successes, de-sensitise) participants to distance, or (2) failures might de-sensitise (successes, sensitise) participants to rewards. We did not hold particular expectations with respect to how guidability might evolve in a reward-dependent manner, because it is unclear whether wins/losses should sensitise or de-sensitise participants to guidability. To implement these models we utilised a very similar update scheme to the one we introduced for the H_t term, with the crucial difference that achievement and failure affect the sensitivity to trial features rather than a separate achievability component. For these models, choice of the vehicle v and goal g at trial t arises as a softmax policy which takes as input the term $U_t(v, g)$ defined below.

For model m_{dist}:

$$U_t(v,g) = -\alpha_d(t) \cdot d_t(v,g) + \alpha_v \cdot 100\gamma_t(v) + \alpha_r \cdot r_t(g) \tag{1}$$

in which the sensitivity to distance (i.e. $\alpha_d(t)$) evolves in a way that depends on reward, and the other α's, weighing guidability (α_v), and reward (α_r) are fixed. Note that we enforce that $\alpha_d(t)$ never falls below zero in our model code.

$$\alpha_d(t) = \alpha_d(t-1) + \begin{cases} 0 & \text{if achieved } g^- \\ -\omega_w & \text{if achieved } g^+ \\ +\omega_l & \text{if lost} \end{cases} \tag{2}$$

Model m_{rew}:

$$U_t(v,g) = -\alpha_d \cdot d_t(v,g) + \alpha_v \cdot 100\gamma_t(v) + \alpha_r(t) \cdot r_t(g) \tag{3}$$

in which the sensitivity to reward evolves in a way that depends on reward.

$$\alpha_r(t) = \alpha_r(t-1) + \begin{cases} 0 & \text{if achieved } g^- \\ +\omega_w & \text{if achieved } g^+ \\ -\omega_l & \text{if lost} \end{cases} \tag{4}$$

In this formulation losses desensitise, while wins sensitise, participants to rewards.
Finally, model m_{veh}:

$$U_t(v,g) = -\alpha_d \cdot d_t(v,g) + \alpha_v(t) \cdot 100\gamma_t(v) + \alpha_r \cdot r_t(g) \tag{5}$$

in which the sensitivity to guidability evolves in a way that depends on reward.

$$\alpha_v(t) = \alpha_v(t-1) + \begin{cases} 0 & \text{if achieved } g^- \\ +\omega_w & \text{if achieved } g^+ \\ +\omega_l & \text{if lost} \end{cases} \tag{6}$$

Note that here ω_w and ω_l are allowed to be positive or negative, whereas in the other two formulations (i.e. m_{dist} and m_{rew}) they are forced to be positive or equal to zero. All models performed worse than our winning model according to WAIC. The table below holds a summary of the WAIC scores obtained. Though we would rather not add these results to the manuscript to keep the already vast set of models as small as possible, we leave it up to the editors and reviewers whether they judge these should be added.
(b) It would be helpful to better understand what your estimate of the RW terms would look like under different generative models (and to validate the discriminability between models with model recovery).

This is a critical point. To address this, we generated 50 datasets from all our models (except model "temporally evolving bias", which we dismissed for simplicity) and performed model recovery analyses on all models. We used the same algorithm as suggested in, for instance Daunizeau, J., Adam, V., & Rigoux, L. (2014). VBA: a probabilistic treatment of nonlinear models for neurobiological and behavioural data. PLoS Comput Biol, 10(1), but using WAIC scores rather than Bayesian model selection. We generated synthetic data from the posterior

Model	WAIC score
Vehicle dep. RW	5118
m_{dist}	5135
m_{rew}	5140
m_{veh}	6391

Table 2: Model comparison for models with outcome-dependent evolution of distance sensitivities (i.e. m_{dist}) and reward sensitivities (i.e. m_{rew}).

parameterisation of each model obtained after fitting to the data. We obtained the confusion matrix as per figure 1. Similar models are at higher risk of being confused – however, our winning model had an expected probability of 0.04 of being wrongfully recovered if a different formulation were true (false positive rate); and a probability of 1 of being recovered if the data were truly generated by it (true positive rate). We added figure 1 here as supplementary information to the manuscript (i.e. Figure S3)).

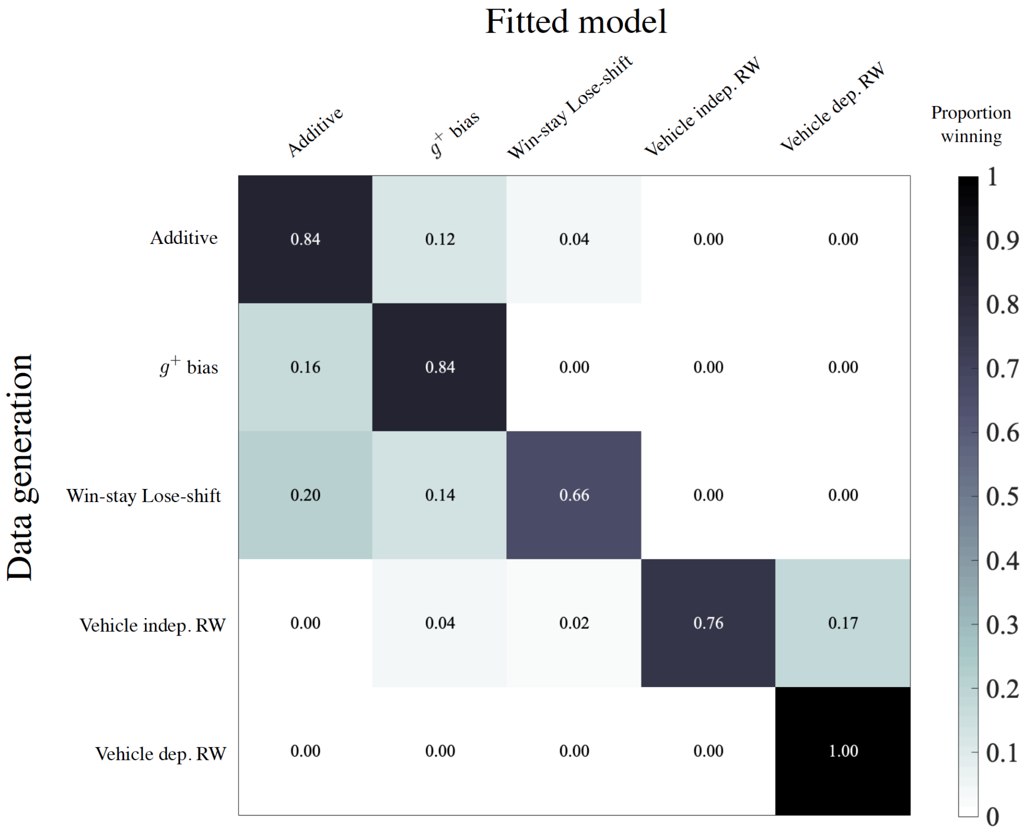

Figure 1: **Confusion matrix for model recovery. We generated 50 datasets from all our models, and performed model recovery analyses on all models (Palminteri et al. 2017), using WAIC scores. We generated synthetic data from the posterior parameterisation of each model obtained after fitting to the data. Similar models are at higher risk of being confused – however, our winning model ("Vehicle dependent RW") had an expected probability of 0.04 of being wrongfully recovered if a different formulation were true (false positive rate); and a probability of 1 of being recovered if the data were truly generated by it (true positive rate).**

In addition, we fit our winning model to obtain the γ parameter so we could compare it with the values obtained when fitting to the original "Vehicle-dependent RW" model. The recovered γ from data generated from models other than the winning model were substantially closer to zero. The posterior distribution of γ when fitting datasets generated by the winning model was in line with the original posterior. See figure 2.

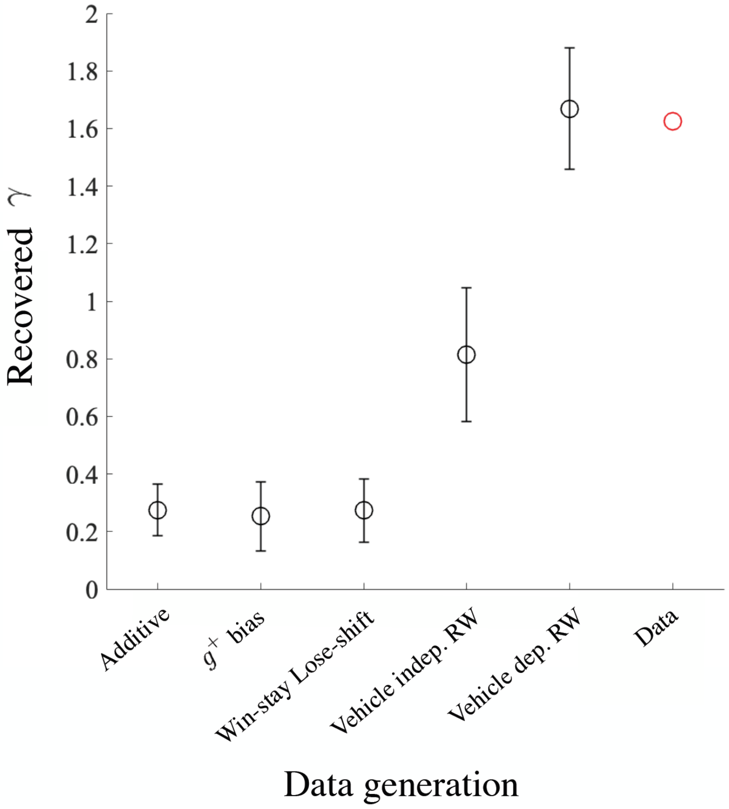

Figure 2: Recovery of the model-free gain (γ) from different generative models. The data is generated by each model under their own posterior parameterisation. Each of 50 datasets is then fit by the winning model, and the recovered posterior medians for γ, for each dataset, are rendered with errorbars (standard deviations of medians of the posteriors obtained from each dataset). When data is generated by models other than "Vehicle dep. RW", the recovered γ's are substantially closer to zero. Values recovered from data generated by "Vehicle indep. RW" are slightly higher, putatively because of this model's similarity to the winning model. The values recovered from data generated by "Vehicle dep. RW" are in line with the original parameterisation (red circle).

(c) Was there any evidence that the vehicle value biased decision in the single-goal phase?

Though we developed an entirely separate class of models to account for SGTs, the reviewer is quite right that we might observe an influence of the RW term towards vehicular preference. To establish whether such an influence might have occurred, we repeated the fit for our best performing SGT model (interaction), adding $H_t(v)$ as per the last double-goal trial (number 16; therefore $H_{16}(v)$). Our augmented winning model is therefore:

$$U_t(v) = -\alpha_d \cdot d_t(v) + \alpha_v \cdot 100\gamma(v) + \alpha_{int} \cdot \gamma(v)d_t(v) + \alpha_H \cdot H_{16}(v) \quad (7)$$

We considered whether this last component would (1) influence decision making, in a way that (2) also reflected

the extent to which term H_t influences decisions in DGTs (i.e. the parameter γ). We first fit the model allowing this coefficient to range in a symmetric interval on the real line, to establish if the fit would yield positive values. A t-test performed on the subject-wise coefficients was significant in high influence conditions ($t(34) = 2.56$, $p = 0.01$) indicating that the distribution of α_H values is larger than 0 - and therefore that the $H_{16}(v)$ term does bias decision-making in SGTs. Further, we found the expected correlation between these coefficients and the model-free gain (i.e. γ) obtained in DGTs ($r = 0.36$, $p = 0.03$), so that the same subjects who weighed the H_t term more during DGTs were more biased towards vehicles holding better achievement records in SGTs. However, neither of these findings was true in low influence conditions, as the t-test could not reject the null hypothesis that the distribution of α_H was centered around zero ($t(34) = -1.49$, $p = 0.14$), and the correlation with γ was non-significant ($r = 0.11$, $p = 0.57$). This is consistent with the fact that in DGTs, γ values take much smaller values in low rather than in high influence conditions, and reflects the impossibility of achieving the goal in single goal trials – which putatively led subjects to noisier decision making.
Nevertheless, the WAIC score of the new interaction model (i.e. equation 7; WAIC: 1273) was in fact better than that of the same model lacking the contribution of the $H_{16}(v)$ term (WAIC: 1282). We therefore decided to re-run all SGTs models adding this term - re-computing WAIC scores for all models. Thus, we modified table 3 in text, which becomes table 3 here.

Model	Description	LOSO	WAIC
1	Additive	0.554	1280
2	Interaction	0.584	1273
3	Additive + Probability	0.543	1286
4	Probability	0.521	1488

Table 3: **Model Comparison for SGTs. The most parsimonious model accounts for data through adding distance and vehicular guidability features, and their multiplicative interaction. I-scores were explicative of distance sensitivities, but only in high influence blocks.**

Point 2. *Just based on this survey, it's hard to make strong claims about internalizing per se, relative to other traits that may be associated with internalizing. A related concern is that this sample size is small for studying individual differences, so influences that one might expect to play a larger role (like mood, SES, education, IQ, OCEAN) may be more likely than a specific LoC subscale.*

(a) The authors should assess the reliability & factor structure for the internalizing scale (and the eigenvalues of the subscale covariance matrix would be more informative than the pairwise correlations).

We performed a VariMax-rotated factor analysis on the single items to obtain the factor structure of the questionnaire responses. We used four components ($p_{\chi^2} = 0.1$). The cumulative variance explained by the first three factors was 35%, in line with the values found in literature (see e.g. Levenson, 1974). We correlated scores (i.e. the projections of the questionnaire responses along the factors) with the I,P and C scales to ascertain whether these were reflected. The results suggest that the first 3 factors largely reflect the 3 dimensions individuated by Levenson (Levenson, 1974). Although the fourth factor appears to individuate a further, non-specific external component (as it is anticorrelated with I-scores, and weakly positively correlated with C and P scores) none of its correlation coefficients reach significance, which suggests that the variability that this dimension captures is likely not enough to warrant a further dimension. In full accord with the reviewers' suggestion, we now substitute

table 1 in text with table 4 below - which reports factor analysis results along with internal consistency measures.

Levenson subscale	Factor loadings				Internal consistency (Cronbach's α)
	factor 1	factor 2	factor 3	factor 4	
**Internality** (I)	-0.47	-0.29	**0.78**	-0.46	**0.73**, 95%CIs $[0.57, 0.88]$
**Chance** (C)	**0.64**	0.40	-0.36	0.28	**0.79**, 95%CIs $[0.67, 0.91]$
**Powerful Others** (P)	0.41	**0.77**	-0.47	0.26	**0.79**, 95%CIs $[0.67, 0.90]$

Table 4: **Analysis of LoC subscales. In this table we report various measures to gain insight into the structure and consistency of the LoC subscales. We performed a varimax-rotated factor analysis on the single items to obtain the factor structure of the questionnaire responses. We used four components ($p_{\chi^2} = 0.1$). The cumulative variance explained by the first three factors was 35%, in line with the values found in literature (see e.g. Levenson, 1974).We correlated scores (i.e. the projections of the questionnaire responses along the factors) with the I,P and C scales to see whether these were reflected (coefficients significant post Bonferroni correction for multiple comparisons are in bold). The results replicate well those found in literature, as they suggest that the first three factors largely reflect the 3 dimensions individuated by Levenson (Levenson 1974). Although the fourth factor appears to individuate a further, non-specific external component (as it is anticorrelated with I-scores, and weakly positively correlated with C and P scores) none of its correlation coefficients reach significance, which suggests that the variability that this dimension captures is not enough to warrant adding a further dimension. Finally, we report internal consistency measures , along with 95% confidence intervals, in the last column.**

(b) The authors should avoid over-interpreting the implications of neuroimaging experiments on LoC based on these results.

We agree with the reviewer's caution. We therefore reworded the discussion to mitigate our interpretation in terms of neuroimaging experiments (p.19), removing most of the discussion from before, and replacing with the paragraph below.

> **In neural terms, the ventromedial prefrontal cortex (vmPFC) is consistently found to be involved in control detection (Kerr et al., 2012; Salomons et al., 2004; Wood et al., 2015; Harnett et al., 2015; Christianson et al., 2009; Wang, 2019). In a study involving LoC, Harnett et al. (2015) found that learning-related changes in the emotional response to negative outcomes were mediated by activity in ventromedial prefrontal cortex (vmPFC); and that, as individuals moved towards the external end of the LoC spectrum, the vmPFC response to predictable threat decreased (the study, however, could not differentiate between internality, chance and powerful others). Our findings could provide a possible, instrumental, amplification of these results, with the suggestion that the rate of learning and adaptation from negative events might be moderated by vmPFC. The vmPFC has duly been implicated in computations involving control detection (Kerret al., 2012; Salomons et al., 2004; Wood et al., 2015; Harnett et al., 2015; Christianson et al., 2009; Wang, 2019), and, in studies in rats, has been shown to suppress the over-exuberant activity of serotonergic neurons in the dorsal raphe when they have control over aversive outcomes (Maier & Watkins, 2005). The latter is one**

of the sources of evidence that serotonin is involved in aversive learning (Daw et al., 2002; Boureau & Dayan, 2011; Dayan & Huys, 2009), and could be involved in the excess learning from failure that we found.

(c) Ideally a replication (e.g. online) would increase confidence in the conclusions and allow other factors to be disentangled.

The task is being deployed at the university of Vienna in different populations with some modifications, and with pharmacological manipulations applied. We thought hard about doing a replication. However, these current results are actually already a replication of a preliminary pilot (which we did not report because of various modest differences), and since we have applied robust and largely conservative statistical methods. Thus, we consider the current results to stand for themselves. If we were to repeat the task, various further changes would be desirable, based on what we have learned here.

point 3. *It would be great if the authors would formalize this decision process in terms of the optimal choice strategy.*

(a) How good are the different vehicle property weighting strategies (additive, multiplicative, RW, etc) at predicting the reward probability?

To answer this question, we first generated the optimal choices for each dataset for each subject, and fit our winning model to the data. Note that we therefore generated only one set of choices, since there exists a best choice for each trial - i.e. one that maximises expected reward. We then fit the winning model to this dataset, thus recovering the parameterisation for this model which best approximates perfect decision making. We plot the posterior distribution obtained below, along with the posterior recovered from actual data, for comparison (figure 3). Note that the initialisation parameter (i.e. H_1) was kept fixed to the original parameterisation obtained from raw data - this is because we were more interested in establishing differences in learning rather than those in initialisation. We can see that the largest differences arise in the distance sensitivity, α_d, in low influence conditions, which would would optimally have been much higher, and the sensitivity to guidability, α_v, which should have been higher in both conditions. Interestingly, In high influence conditions, the learning gain would have optimally been much lower (parameter α_H), indicating that a higher sensitivity to trial features alone (esp. the objective guidability of vehicles) would have led to better performances. The learning rates from failure, ϵ_l, should have been higher too.

(b) The conceptual differences between influence and guidability are interesting, and could be further elaborated. How should choice algorithms use these two sources of information (e.g,. How would they alter the transition matrix of a model-based controller)?

Guidability and influence are two facets of the concept of controllability. In our task, guidability is inherent to a vehicle; whereas influence determines how benign the environment is in allowing subjects to make many effective presses. In this circumstance, high controllability requires both high influence and guidability. One key observation is that the poorer the influence the more important guidability becomes, and the poorer the guidability the more important influence becomes. Because we have set up such a stark difference in influence

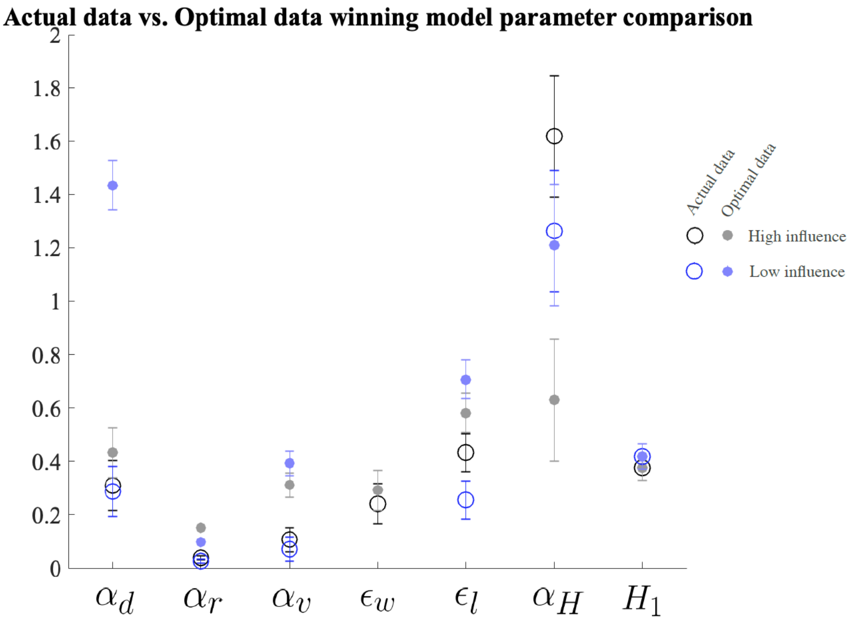

Figure 3: Comparison of recovered parameters on data from an optimal decision maker and subjects. Note that the H_1 term was kept fixed to the subjective values obtained from data. This is because we thought it would be more important to focus on the sub-optimality of learning rather than in the initialisation parameter - since this likely arises as a consequence of early training trials.

across conditions, we unfortunately cannot address how subjects trade between the two.

The title is uninformative - would be more helpful if it summarized the main finding.

We have changed the title to: "Internality and the Internalisation of Failure: Evidence From a Novel Task".

Reviewer 3

In this paper, the authors have designed a new task in which participants choose between different tools (cars) to reach various simple goals (targets leading to rewards - punishment if they don't reach any goal). The cars are only partially reliable (noisy) and intermittently effective (the effort exerted by the participant has more or less influence) to degrees that need to be learned. The authors are interested in the relationship between participants' scores related to locus of control/ internality with respects to self (Levenson I-scores) and their performance at the task (in particular whether participants choose the more rewarding goal which is taken as a read out of their subjective tradeoff between achievability and reward). Computational analyses showed that the negative relationship between the most rewarding goal choice frequency and I-scores was explained by boosted learning from failure and biased initialisation of achievability.
I found this paper, and more generally the question regarding subjective perception of controllability, very interesting and the methodological aspects are novel, original and solid. The paper will be inspiring for the computational psychiatry community.

We thank the reviewer for the kind words.

However, I think the paper (and abstract) could be reworked to make it a clearer and more impactful read. The questions asked, their significance, the hypotheses and importance of the results could come across in a clearer way. Similarly the logic behind the design of the task could be clearer, e.g. it is initially unclear whether the authors are interested mostly in influence of initial expectations about control, aspects of learning or decision-making, what the hypotheses are with respect to the questionnaires (what patterns of behaviour would be expected under different hypotheses and are important to distinguish) and how they could generalise to more meaningful situations.

To address this comment, we re-wrote the introduction and methods to describe explicitly our hypotheses and expectations. The following section, from the new introduction is particularly relevant to the comment:

> [...]**More specifically, our manipulations are aimed at resolving how internality (I-scores in the LoC scale; Rotter, 1966) and beliefs in the influences of Luck (C-scores) (1) affect the choice between plans offering various trade-offs of macroscopic controllability and reward, (2) are manifest in explicit biases towards either macroscopic or microscopic scales of controllability, and finally (3) affect the way outcomes (in the form of success and failure to achieve a goal) guide the learning of controllability and, in turn, decision making. Previous work touching on these issues is limited (see e.g. Julian et al., 1968; Strickland Rodwan, 1964)- and we know of no previous paradigm in which subjects had to learn controllability as they performed actions to reach goals. The study that perhaps comes closest to this Julian et al. (1968), who reported a dart throwing paradigm in which subjects first estimated the two distances from which they could score with five and seven darts respectively (this step equates the macroscopic controllability of the two options), and were subsequently asked to choose the one distance from which they would prefer to throw (i.e. five darts from the closer, and seven darts from the farther distance). The closer distance offers higher microscopic controllability, since each single throw has a higher probability of scoring – and this is the option that internals were found to prefer. However, by design of the paradigm, (1) subjects do not learn from their own performance, and can not revisit their choices based on their successes and failures, and (2) the trade-off with macroscopic controllability is not manipulated (what if, for instance, subjects were given more darts to throw from the farther distance - how many more darts would it take to switch the preference to the farther set of tries?) so that we can not examine the influences of attribution on learning, and the tradeoff between macro- and micro- scopic controllabilities. We sought to design a more comprehensive paradigm addressing these issues,and allowing us to examine how interactions among prior expectations, attributions, and learning, affect choice and then performance.**[...]

The relevance and application of the results to mental illness could also be clarified (e.g. are the questionnaire scores relevant to dimensions such as low mood? anxiety? do the results have implications for psychotherapy?).

We agree, and to answer this comment, we added a paragraph in the introduction that touches on the psychological relevance of LoC:

> **Though LoC is usually thought of as a form of personality orientation, external loci are a common co-occurring trait in various disorders - and shifts towards internalization are often a consequence of treatment (Frank et al., 1978, p.42). In a meta-study,Presson & Benassi (1996) found solid relationships linking external orientations with depression severity, as measured by a variety of questionnaires (e.g. BDI); and generally, the number of studies which evidence an inverse relationship between locus of control and psychiatric symptoms is quite large (e.g. Johnson & Sarason, 1978; Solomon et al., 1988; Hoehn-Saric & McLeod, 1985**

We also added a paragraph in the discussion, briefly addressing the possible clinical ramifications of the results:

> **From a clinical perspective, our results point to the successful integration of negative information about the self (failure) as a prominent factor for internality, and point towards a computational trait (learning from failure) which has the virtue of guarding subjects from loss, and subserves the maintenance of a healthy idea of controllability, and might be lacking or weak in many disorders. Our observations are then relevant to those disorders in which the integration of aversive information would be a requirement for alleviating symptoms – such as those characterised by perseverative dysfunctional thoughts or behaviours. However, it is only through further applications of our task in clinical populations that we will gain more insights into these possibilities.**

Regarding the results, it is also unclear initially whether the results could simply be interpreted in terms of high-I participants preferring more controllable choices (after having learned which are controllable - which would sound like a somewhat trivial result).

The reviewer is right that the model-agnostic section of our results highlights that I-scores correlate with the rate of more controllable choices (i.e. they anticorrelate with choice of g^+ goals). However the computational analyses suggest that it is the speed at which they learn controllability which varies across the spectrum of internality (i.e. internals learn faster about the inachievability of g^+ goals following failure). Thus, for instance, all subjects will eventually learn that g^+ goals are less achievable than they first thought – but the speed at which this process takes place is the factor relating to internality. Thus, while the first result is expected, the second result is the one that actually points to a computational trait.

I was also wondering how the authors' results could be interpreted in terms of / are controlled for risk and loss aversion (and possibly anxiety) ...

The reward sensitivities appear to play a very minor role in our task (the reward sensitivities are really small compared to other parameters; see Figure S2, Supplementary Information), implying that decision making mostly takes place based on the perception of factors of controllability (i.e. distance, guidability and influence). Further, the learning rates, and not a static feature of decision-making, are the parameters most strongly underlying the model-agnostic effect of reduced propensity to choose g^+ goals, which makes it unlikely that a static feature of decision making (such as sensitivity to losses, or risk - i.e. outcome uncertainty) would play a

more important role. Nonetheless, a shortcoming of our task is of course that we only present subjects with a fixed money detraction (-0.15£) for failure, so that sensitivities to losses can not be compared to sensitivities to wins. This is going to be changed in future versions of the task. We point to this issue in the Discussion:

> **[...]From a design perspective, it would be desirable to have a range of different losses, so that sensitivity to negative outcomes could be assessed [...]**

...and what was the rationale for not using potentially relevant different questionnaires in addition to locus of control.

This is a very important point, and we are aware that more questionnaire measures could have been used to rule out other influences than control beliefs. This being the first experiment to use this task, we only focused on our initial hypotheses on control. Future experiments will deploy a selection of other questionnaires to disentangle other factors.

Some aspects are confusing for e.g. in the abstract and some places in the text "High internality (Levenson I-score) was associated with decision making patterns that are less vulnerable to failure, and at the same time less oriented to more rewarding achievements." If I understand correctly, the latter result (less oriented to more rewarding achievements) is highly dependent on the design of the task (where choosing the more rewarding goal is by design less likely to lead to success). It seems to me that it would be important to distinguish results that are general and potentially meaningful in wider contexts, from those that are specific to the task design, otherwise the take home messages become unclear.

We agree with the reviewer, we therefore made an edit to the first paragraph of the discussion to reflect this:

> [...]In the high influence condition, the timidity of high I-scorers entailed that they had a higher likelihood of success (albeit not gaining more points on average, **because this meant ,by design, that they chose the impoverished goals**).[...]

We also removed a sentence from the abstract:

> [...]High internality (Levenson I-score) was associated with decision making patterns that are less vulnerable to failure~~, and at the same time less oriented to more rewarding achievements~~[...]

- In Author summary: "Our findings can be interpreted within a theoretical framework which implicates control expectations in individual learning differences, in a way that resonates with perseveration and also fit well within modern theories of learning in aversive contexts and serotonergic function. " In absence of further explanation, I found that sentence unclear.

We updated the author summary, removing part of it, i.e.

> [...]Our findings can be interpreted within a theoretical framework which implicates control expectations in individual learning differences, ~~in a way that resonates with perseveration –~~ and ~~also~~ fit well within modern theories of learning in aversive contexts and serotonergic function [...]

We retain the rest of this sentence because we briefly motivate in the discussion why we think our results fit well within modern theories of learning in aversive contexts and serotonergic function.

1 Reviewer 4

This is a well written manuscript on an important matter. I read this manuscript with great interest and I very much appreciate the task design and efforts with the computational procedures. I have some major and minor comments for the authors, they should consider for revising this manuscript.

We thank the reviewer for the kind words.

Comment 1: *While the task design for itself is clever, I am not sure whether the authors have sufficiently embedded the task in the existing body of literature and theory on the concept of control. This is rather vaguely justified in the introduction with the statement "Thus, we lack a task that decomposes objective controllability into its finer parts described above, and assesses the interaction between outcomes, ascription style, expectations and choices." What exactly are the finer parts described above? At least for me, this is unclear and more importantly I cannot find a clear description on how these map on the current task design and the specific model parameters that are used to explain behavior. I think the specifics of the task design need to be much more thoroughly and clearly integrated to the existing literature so one can understand the rationale for why this task is needed and useful to understand an important aspect of controllability. More precisely, which aspects of the parts of controllability do the manipulations of "influence" or "guidability/reliability/contingency" address that have been brought up as relevant in the literature? Or are these just necessities for examining learning? Why are the different "influence" conditions needed? I very much believe that this task merits a more profound introduction and justification here.*

To address this comment, we have extensively re-written both introduction and methods, to provide a reference to the literature and exemplify and detail the manipulations that we use. In the introduction, we try to be more specific about the finer parts of control which we aim to address, and also make a reference to a particularly relevant study:

> **[...]More specifically, our manipulations are aimed at resolving how internality (I-scores in the LoC scale; Rotter, 1966) and beliefs in the influences of Luck (C-scores) (1) affect the choice between plans offering various trade-offs of macroscopic controllability and reward, (2) are manifest in explicit biases towards either macroscopic or microscopic scales of controllability, and finally (3) affect the way outcomes (in the form of success and failure to achieve a goal) guide the learning of controllability and, in turn, decision making. Previous work touching on these issues is limited (see e.g. Julian et al., 1968; Strickland & Rodwan, 1964)- and we know of no previous paradigm in which subjects had to learn controllability as they performed actions to reach goals. The study that perhaps comes closest to this Julian et al. (1968), who reported a dart throwing paradigm in which subjects first estimated the two distances from which they could score with five and seven darts respectively (this step equates the macroscopic controllability of the two options), and were subsequently asked to choose the one distance from which they would prefer to throw (i.e. five darts from the closer, and seven darts from the farther distance). The closer distance offers higher microscopic controllability, since each single throw has a higher probability of scoring – and this is the option that internals were found to prefer. However, by design of the paradigm, (1) subjects do not learn from their own performance, and can not revisit their choices**

based on their successes and failures, and (2) the trade-off with macroscopic controllability is not manipulated (what if, for instance, subjects were given more darts to throw from the farther distance - how many more darts would it take to switch the preference to the farther set of tries?) so that we can not examine the influences of attribution on learning, and the trade-off between macro- and micro- scopic controllabilities. We sought to design a more comprehensive paradigm addressing these issues,and allowing us to examine how interactions among prior expectations, attributions, and learning, affect choice and then performance.[. . .]

In the methods section, we cover more systematically and in a more detailed manner the control manipulations used. We turn the previous subsection (i.e. "Control manipulations") into an entirely refurbished subsection titled "Rationale of the design and control manipulations", which we report below:

3.4.1 Rationale of the design and control manipulations.

The gross probability of attaining a goal in our task depends on (a) the *distance* separating the vehicle from the goal - a significant contributor to the macroscopic controllability of that goal (i.e. the overall probability of reaching the goal); (b) the *guidability* of each vehicle, by which we operationalise microscopic controllability; and (c) the *influence* that subjects can exert, which sets the efficacy of effort.

We parameterised the guidability of the vehicles in terms of conditional probability (Huys & Dayan, 2009): independently, at each press, the vehicle follows the subject's intended direction (i.e., the chosen arrow key) with probability γ, or moves in a random direction (drawn uniformly) with probability $1 - \gamma$ (note that this includes the arrow direction followed by chance). For a certain pressing frequency, the true probability of success depends both on how (1) distant from the goal, and (2) guidable the vehicle is, and is nearly always (except for trial type OG in double goal trials) orthogonal to the amount of reward that the goal offers - so that the sensitivity to macroscopic controllability can simply be measured as the proportion of choices to attain the less rewarding goal. Analogously, microscopic control sensitivity can be measured as the proportion of choices of the more controllable of the two vehicles. Subjects preferring lower rewards are more sensitive to macroscopic control than reward (since, in DGTs, distances and reward amounts are orthogonal); conversely, subjects preferring more rewarding goals regardless of the vehicle used neglect either form of control; and finally, subjects preferring more controllable vehicles are more sensitive to microscopic control. The manipulation of influence is perhaps the most restrictive - as it sets a hard limit on the maximum number of steps per second that vehicles would move. We signaled two types of block: a benevolent type ("high influence") in which this limit was set to 8 steps per second ($8Hz$), and a malevolent type ("low influence") in which this limit was set to $4Hz$. Note that all subjects could reach (and in many cases, break) the maximum pressing frequency of $8Hz$. In high influence blocks, higher pressing frequencies are designed to entail higher rewards, but in low influence blocks, the two are uncorrelated. We introduced this manipulation to test whether influences

> **of internality on behaviour would be more salient in inherently benign or malignant environments (i.e. environments in which higher efforts lead to higher rewards on average, and in which they do not, respectively).**
>
> **In order to maximise expected rewards, subjects should approximate the probability of reaching the goal with a certain vehicle, combining distance and guidability information (i.e. how likely a vehicle with a certain guidability is to cover the distance separating it from the goal in a timely manner). In forming this approximation of probability, subjects will not just use trial features such as distance and guidability – their choices will be informed by the history of achievement and failure (which is subject to influence), and thus on their subjective attribution schemes. For instance, if I failed to reach the goal with a certain vehicle from a certain distance, the question of whether I will try again should at least in part be informed by my prior experience of failure. As we will see, our task also allows disentangling such history-dependent influences through simple model-based analyses.**

In this line, the summary from l 45 on is somewhat difficult to understand based on the brief description. The "reliability" at the beginning refers to the "contingency" later but I would recommend introducing one concept – as has been done with influence - and keep refereeing to it in the following. Later in the manuscript you use guidability. I am honestly not sure which concept best differentiates the two components of your design, but I would introduce something here that you explain and stick to.

To address this issue, we re-wrote the part of the introduction in which we offer a precis of the design, immediately introducing and clarifying the relevant concepts so that it is easier to follow the full description of the task in methods.

> [. . .] **Here, we present a more ecological task intended to separate different components of controllability, allowing us to discriminate the subjective sensitivity to some of its micro- and macro-scopic aspects, and their interaction with outcomes, ascription style, expectations and choices. In our task, subjects choose between, and then use, one of a range of tools to achieve various simple goals. The tools are vehicles (as in our example of the bike), and are only partially reliable (with differing amounts of entropy in the effects of choices; a quantity we will refer to as guidability) and intermittently effective (affording many or few actions per unit interval; we call this influence), to degrees that need to be learned. It is the requirement for execution that makes the subjects' achievements depend at least partly directly on their microscopic behaviour, and, as above, that makes the learning of the probability of achievement dependent on ascription style (did I fail because I did not press hard enough? did I fail because of bad luck?).**[. . .]

Comment 2: *I fear that this study might not be sufficiently powered, or that the expectations the authors are having with regards to the underlying effect size is too optimistic. That is a limitation of the study, that should be discussed. .45 is quite a large effect for correlational measures and this study is even underpowered for medium effects. On which ground do you assume that the associations you are looking for might be this large?*

We agree, and we point to this issue in the discussion:

> **[...]Despite these promising findings, we should note some caveats which concern our results. For instance, while we found no significant relationships involving the Chance sub-scale, it is entirely possible that these might exist, but simply play less apparent roles than the I- (or P-) sub-scales. Replications of our study, possibly availing of larger sample sizes, could offer further support to our results.[...]**

On a related note, I was wondering if the authors have considered that they always tested two scales and corrected for the associated increased Type 1 error.

Yes - we use correction for false discovery rates throughout the study. In our model agnostic analyses our results are always protected for using both the I, and C, scales. Since the model-dependent analyses are meant to offer insight into the model-agnostic effect, we already rule out relationships with the C-scale, so our set of hypotheses only concerns the relationship between the I-scale and the model parameters.

Comment 3: ll278 – 285 (i) *Would you be so kind a provide the report of the results identical for the high and low influence blocks. Your report for the high-influence block is more complete compared to the low influence block without further explanation. Also, if you find no significant relationship between the amount of money earned and I-scores, please provide the relevant statistics for this.*

We appreciate the importance of this omission, and have edited the portion of text highlighted by the reviewer, reporting identical statistics for both influence conditions:

> **[...] Conversely, in low influence blocks, we did not observe a correlation between I-scores and probability of success (r = 0.19, p=.24 95%CIs [-0.15, .49]), or the correlation between the amount of money earned and I-scores (r = -0.11, p=.43 95%CIs [-0.44, .20]); this is possibly because in low influence blocks the margin to make safer decisions is drastically reduced: all probabilities of success for all options are lower.[...]**

(ii) *Related to this, I was wondering if the associations significantly differed between the two influence conditions and whether this would be important for your set of hypotheses to test? If you want to discuss findings as if they differ between the conditions, you should also test it here, if I understand correctly (as you e.g. do in l 404). Please watch carefully, that if you want to discuss associations to differ between conditions, as being smaller or stronger, that you also test the difference, because otherwise this claim is not backed up by statistical inference.*

The associations did not differ between the two influence conditions, however this did not belong to our set of hypotheses. The two influence conditions are only there to test whether different strategies would be put in place according to the overall benignity of the environment, and to test whether factors that are potentially relevant in one particular influence condition are also relevant in the other. For instance, in SGTs, the relationship between LoC and sensitivity to distance could only be detected in high influence conditions. We realise that this must be confusing, so to not puzzle the reader, we remove the parts in text where we underline that the effects were stronger in low influence conditions; i.e. in the caption of figure 5:

> [...] ~~The effect is slightly stronger in low influence conditions~~ [...]

and in discussion:

> [...] This correlation was significant when subjects had either greater or lesser influence over the environment, ~~but was stronger in the latter condition~~. [...]

Comment 4: *I am not sure if I would agree to your evaluation of the results in ll 292-294: "This suggests, on a model-agnostic perspective (though this will be examined in more detail using modeling), that the microscopic action-to-outcome noise (or more abstractly, the objective quality of the tool used to carry out the plan) is not a particularly prominent feature of controllability in this task." As far as I understand it is just not significantly related to individual differences in the two facets of controllability, but that does not imply it is not a prominent feature of the task. It might be extremely relevant so that anybody, whether experiencing high or low control in everyday live, would pick the tool with better quality. I would suggest rephrasing this section.*

We completely agree with the reviewer, and rephrased our interpretation:

> [...] This suggests, on a model-agnostic perspective (though this will be examined in more detail using modeling), that the microscopic action-to-outcome noise (or more abstractly, the objective quality of the tool used to carry out the plan) **is not related to individual differences in LoC I- or C- scores** [...].

Comment 5: *Could you provide internal consistency measures for the LOC scales?*

Yes, we now report these in table 1 in text, which we add here for convenience (table 5 below, last column).

Levenson subscale	Factor loadings				Internal consistency (Cronbach's α)
	factor 1	factor 2	factor 3	factor 4	
Internality (I)	-0.47	-0.29	**0.78**	-0.46	**0.73**, 95%CIs $[0.57, 0.88]$
Chance (C)	**0.64**	0.40	-0.36	0.28	**0.79**, 95%CIs $[0.67, 0.91]$
Powerful Others (P)	0.41	**0.77**	-0.47	0.26	**0.79**, 95%CIs $[0.67, 0.90]$

Table 5: **Analysis of LoC subscales. In this table we report various measures to gain insight into the structure and consistency of the LoC subscales. We performed a varimax-rotated factor analysis on the single items to obtain the factor structure of the questionnaire responses. We used four components ($p_{\chi^2} = 0.1$). The cumulative variance explained by the first three factors was 35%, in line with the values found in literature (see e.g. Levenson, 1974).We correlated scores (i.e. the projections of the questionnaire responses along the factors) with the I,P and C scales to see whether these were reflected (coefficients significant post Bonferroni correction for multiple comparisons are in bold). The results replicate well those found in literature, as they suggest that the first three factors largely reflect the 3 dimensions individuated by Levenson (Levenson 1974). Although the fourth factor appears to individuate a further, non-specific external component (as it is anticorrelated with I-scores, and weakly positively correlated with C and P scores) none of its correlation coefficients reach significance, which suggests that the variability that this dimension captures is not enough to warrant adding a further dimension. Finally, we report internal consistency measures , along with 95% confidence intervals, in the last column.**

Comment 6: *Figure 5. I would recommend removing the sentence "The effect is slightly stronger in low influence conditions." At the end of the caption. Numerically that might be true, but this tiny difference is not*

meaningful. It seems not to be contributing to an argument anyway, doesn't it? Also, I would advise to use it only if you tested the associations against each other also in the following figures and analyses.

We agree, and have removed the sentence.

Comment 7: *Figure 7. I was wondering why you use two different correction procedures for multiple comparisons and highlight these differently in the figure. I would suggest using either Bonferroni or FDR. The different number of stars for the different measures do not seem to make sense. Also, the legend is not easy to understand. The terms Necessary + predictive (why in italics) are only explained in the caption. I think you can make it easier for the reader to understand what is going on her and exactly clarify what necessary and predictive means.*

To answer this comment, we give further explanation on the necessity of using analyses for necessity and predictiveness in methods:

> [...]**The rationale underlying the use of these measures to establish (1) necessity for recovery of the model agnostic effect and (2) predictiveness of the parameters in relation to LoC-scores is that the former is helpful in understanding which parameter underpins the model-agnostic effect found, while the latter by-passes the choice of a model-agnostic outcome measure and provides a way of mapping the recovered parameters onto LoC.** [...]

We also change the figure to remove the italic in the legend, and change the caption to reflect the fact that we only use fdr correction for multiple comparisons (see figure 4).

Comment 8: *There seems to be missing a "what" in l 6 "... or affecting prior notions about exactly is achievable or what we can or cannot do."*

We thank the reviewer for spotting this - we added the missing word in text.

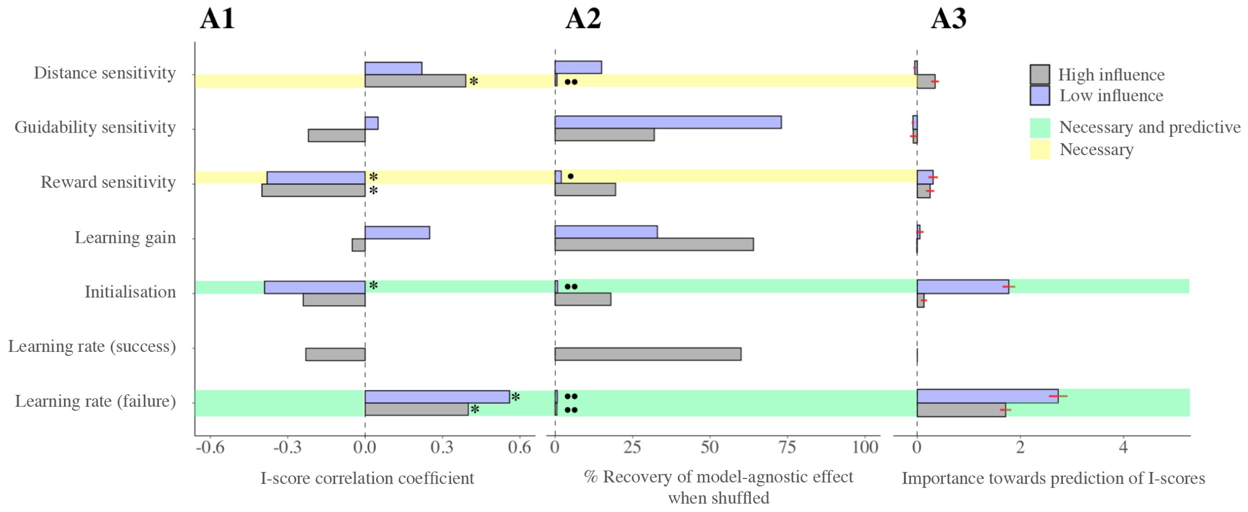

Figure 4: **I-scores and parameter estimates. This series of plots illustrates the relationship between I-scores and the recovered parameter estimates of the winning model (i.e. 'Vehicle dependent RW', in table 2) in three ways. Note that learning rate from success (in attaining g^+) is absent in low influence conditions, as only 3 wins over all datasets were recorded. A1 illustrates the correlation coefficients between each parameter and I-scores. Next to each bar, a star (*) indicates significant correlations post false discovery rate correction for multiple comparisons. ~~Two stars (**) indicate significant correlations post Bonferroni correction for multiple comparisons.~~ In A2, each bar exemplifies the percentage of recovery of an equal or larger correlation coefficient of I-scores and g^+ choice frequency (the effect found in model-agnostic analyses) when shuffling the recovered parameters across subjects. We performed this analysis (described in methods) to understand which parameters played the most important role in driving this effect. Two circles next to the bar indicate a percentage of recovery smaller than 1%, one a percentage smaller than 5%. Thus, according to this measure, the effect is driven by distance sensitivities (the most apparent feature of achievability) in high influence conditions, and (in both influence conditions) by the learning rates from failure. Finally, in A3, each bar shows the loss in predictive power of I-scores when using the recovered parameter estimates as predictors. This measure used random forests, and was obtained as described in Methods. The errorbars signify the standard errors (the procedure was repeated 50 times). Finally, as the legend shows, the parameters highlighted in green are those which show significant effects with I-scores, are needed to reproduce the model agnostic effect, and exhibit a very high importance for I-score prediction. In yellow, we highlighted those parameters which, albeit being necessary to reproduce the model-agnostic effect, turned out not to be critical for predicting I-scores.**
