# Supplementary material for: Internality and the internalisation of failure: Evidence from a novel task
Source: PLoS Comput Biol. 2021 Jul 6;17(7):e1009134. doi: 10.1371/journal.pcbi.1009134 (PMC8284820; doi:10.1371/journal.pcbi.1009134)
Supplement: Attachment — Submitted filename: ResponseToReviewers.pdf [file pcbi.1009134.s012.pdf]

Response to reviewers

April 13, 2021

Dear reviewers,

We are very grateful for your further efforts reviewing our manuscript, 'Internality and the Internalisation of Failure: Evidence From a Novel Task'. Please find our response below, with original comments by each reviewer in cursive fonts, our response to each comment in normal fonts, and changes to the manuscript in bold fonts. When contents of tables, figures, or equations, are changed, we will explicitly provide a reference to the corresponding element in text. All changes have been added to the document using red font, so that these are identifiable.

Reviewer 1

We have now produced an improved version of our materials in the form of an R notebook. This along with other relevant material are enclosed in the folder `reproducibility_pilot_materials`. We have ensured that all code can generate the paper figures correctly.

Reviewer 2

The authors have made a substantial effort to incorporate the reviewers' feedback into their manuscript, which we believe has improved the paper by further validating the relationship between their task and model. While we believe that this is a strong paper about a novel approach to studying complex decision making, their validations have raised some further questions about their best-performing model, that should be addressed in order to have full confidence in their conclusions.

Thank you for these kind words. We appreciate your further points and have addressed them all.

Point 1. *We commend the authors for testing whether the participants are learning the value of individual features in the task. However, the feature-learning we had in mind involved learning the value of multiple features simultaneously, instead of the heuristic approach of just tracking a single feature. Given that two of the 1-feature RW models would be the second and third best performing models, it's worth investigating whether participants learn the value of multiple features.*

(a) The authors should test whether 2- or 3-feature RW models out-perform the vehicle-level RW model.

As suggested, we tested four further models in which subjects learned the value of multiple features in a reward-driven manner. All models performed worse than our winning model according to WAIC. We added results pertaining to this approach in SMs (Supplementary Text S3) which we report below.

Feature-based learning models. **To determine whether participants impute outcomes to individual trial features, rather than vehicle value (i.e. the term H_t in our winning model), we evaluated an alternative class of models in which subjects' sensitivities to individual trial features evolve over time in a reward-driven manner. The sensitivities concerned are those to (1) distance (model m_d), (2) reward (model m_r) and (3) guidability (model m_v). These models posit in particular that (1) failures might sensitise (and successes, de-sensitise) participants to distance, or (2) failures might de-sensitise (and successes, sensitise) participants to rewards. We did not have particular expectations with respect to how guidability might evolve in a reward-dependent manner, because it is unclear whether wins/losses should sensitise or de-sensitise participants to guidability (i.e. guidability is a feature that is important both for achieving goals and avoiding failures).**

To implement these models we utilised a very similar update scheme to the one we introduced for the H_t term, with the crucial difference that achievement and failure affect the sensitivity to trial features rather than a separate achievability component. For these models, choice of the vehicle v and goal g at trial t arises as a softmax policy which takes as input the term $U_t(v, g)$ defined below. For model m_d:

$$U_t(v, g) = -\alpha_d(t) \cdot d_t(v, g) + \alpha_v \cdot 100\gamma_t(v) + \alpha_r \cdot r_t(g) \tag{1}$$

in which the sensitivity to distance (i.e. $\alpha_d(t)$) evolves in a way that depends on reward, and the other α's, weighing guidability (α_v), and reward (α_r) are fixed. Note that we enforce that $\alpha_d(t)$ never falls below zero in our model code.

$$\alpha_d(t) = \alpha_d(t-1) + \begin{cases} 0 & \text{if achieved } g^- \\ -\omega_w & \text{if achieved } g^+ \\ +\omega_l & \text{if lost} \end{cases} \tag{2}$$

Model m_r:

$$U_t(v, g) = -\alpha_d \cdot d_t(v, g) + \alpha_v \cdot 100\gamma_t(v) + \alpha_r(t) \cdot r_t(g) \tag{3}$$

in which the sensitivity to reward evolves in a way that depends on reward.

$$\alpha_r(t) = \alpha_r(t-1) + \begin{cases} 0 & \text{if achieved } g^- \\ +\omega_w & \text{if achieved } g^+ \\ -\omega_l & \text{if lost} \end{cases} \tag{4}$$

In this formulation losses desensitise, while wins sensitise, participants to rewards.
Finally, model m_v:

$$U_t(v, g) = -\alpha_d \cdot d_t(v, g) + \alpha_v(t) \cdot 100\gamma_t(v) + \alpha_r \cdot r_t(g) \tag{5}$$

in which the sensitivity to guidability evolves in a way that depends on reward.

$$\alpha_v(t) = \alpha_v(t-1) + \begin{cases} 0 & \text{if achieved } g^- \\ +\omega_w & \text{if achieved } g^+ \\ +\omega_l & \text{if lost} \end{cases} \tag{6}$$

Note that here ω_w and ω_l are allowed to be positive or negative, whereas in the other two formulations (i.e. m_d and m_r) they are forced to be positive or equal to zero. These models posit the learning of a single feature. To establish whether subjects might have been learning the value of multiple features at once, we simply combined the learning schemes above. There were then three models in which subjects learned about 2 features (m_{d+r}, combining distance and reward value learning; m_{d+v}, combining distance and guidability value learning; and finally m_{r+v} combining guidability and reward value learning). One last model learned about all features (i.e. m_{d+r+v}).
All models performed worse than our winning model according to WAIC. The table below holds a summary of the WAIC scores obtained.

Model	WAIC score
Vehicle dep. RW	5118
m_d	5135
m_r	5140
m_v	6391
m_{d+r}	**5214**
m_{d+v}	**5130**
m_{r+v}	**5223**
m_{d+r+v}	**5216**

Table 1: Model comparison for models with outcome-dependent evolution of distance (i.e. m_d), guidability (m_v), reward sensitivities (i.e. m_r), and their combinations.

In text, we point the reader to this approach in the results section:

> [...]**Finally, after having established that learning models performed better (i.e. models 3,4, and 5), we went on to test a further class of models in which subjects learned about the values of (subsets of) task-relevant features (i.e. distance, guidability and reward) simultaneously. We report details of the results on this approach in Supplementary Text S3. For now, we just note that none of these models achieved a better performance than "Vehicle dependent RW", though some performed quite well.** [...]

(b) Given the strong performance of even 1-feature RW models, the authors should consider reporting this approach in the manuscript.

We agree with the reviewers and now report on this approach in the manuscript. We quote the relevant sections in our answer to the point above.

Point 2. *We need some clarification on the author's implementation of the ideal observer analysis. Our understanding of the optimal behavior is that it should be closest to the additive model (e.g., the RW models are framed*

as model-free), for which you have low confusability with the RW models. However, it appears that the RW fit to the ideal observer produces substantial vehicle-learning components. If our understanding is correct, this may have several important implications for how we understand these learning models. The authors should try to understand why the ideal observer appears to use a model-free learning strategy, including the recommendations below. It may also be the case that the optimal strategy involves the use of vehicle-level learning. If this is true, the authors would do well to understand why this is the case, and ought to re-frame these learning processes as part of the optimal decision strategy, instead of a model-free heuristic as they currently present in the text.

This is indeed an important point. Optimal decision-making in our task requires knowledge of the probability of reaching the goal so as to be able to compute the expected reward of choosing a vehicle to reach a certain goal. As shown in supplementary materials, a good *approximation* of this probability requires multiplicative interactions, and nonlinear transformations, of vehicular controllability and distance from the goal. Simple linear transforms of vehicular controllability and distance such as the ones we embed in our additive model (i.e. α_v and α_d) are incapable of supporting a faithful estimate of the probability of achievement. By contrast, the model-free heuristic in our winning model offers the computational shortcut of learning the probability of reaching the goal based on the subjective history of success and failure. We believe this to be the reason why fitting the winning model to the ideal observer produces substantial vehicle-learning components. Of course, the model-free heuristic would still be slower to learn than full model-based control that depends on knowing all aspects of the rules of the task.

(a) It could be that there are parameter trade-offs that allow RW components to mimic other parameterizations. The authors should perform parameter recovery on their winning model to ensure that they can accurately identify the contributions of the RW learning parameters.

We performed parameter recovery by generating, and then fitting, synthetic data (150 synthetic datasets) from our winning model. The results are illustrated in figure 1 and caption in this reply. We added this figure to the manuscript as Supplementary Figure S5. This analysis showed that it is hard to do recovery well in low influence conditions, as the initialisation parameter can be somewhat confused with the learning rate from failure. This means that in these conditions, the fact that both learning from failure and biased initialisation are predictive of I-scores likely stems from the fact that we can not disentangle these from data, rather than from the fact that both are involved.

By contrast, high influence conditions offered a cleaner link between the original and recovered learning rates from failure (recall that this is also the condition in which we found that vehicle value biased decision making in SGTs) - so we can gain further confidence in our conclusions about learning from this particular condition.

To inform the reader about the potential confound in low influence conditions, we added the following in the results section:

> [...] **our parameter recovery analyses found a measure of confusion between parameters H_1 and ϵ_l in low influence conditions (Figure S5). This is likely the reason that both are involved in predicting I-scores, and stems from the fact that learning of unachievability happens very quickly as influence is so greatly compromised. This speed of learning means that there are too few trials to disentangle biased initialisation from fast learning. However, in high influence conditions, learning rates are well recovered and are thus**

identifiable. [. . .]

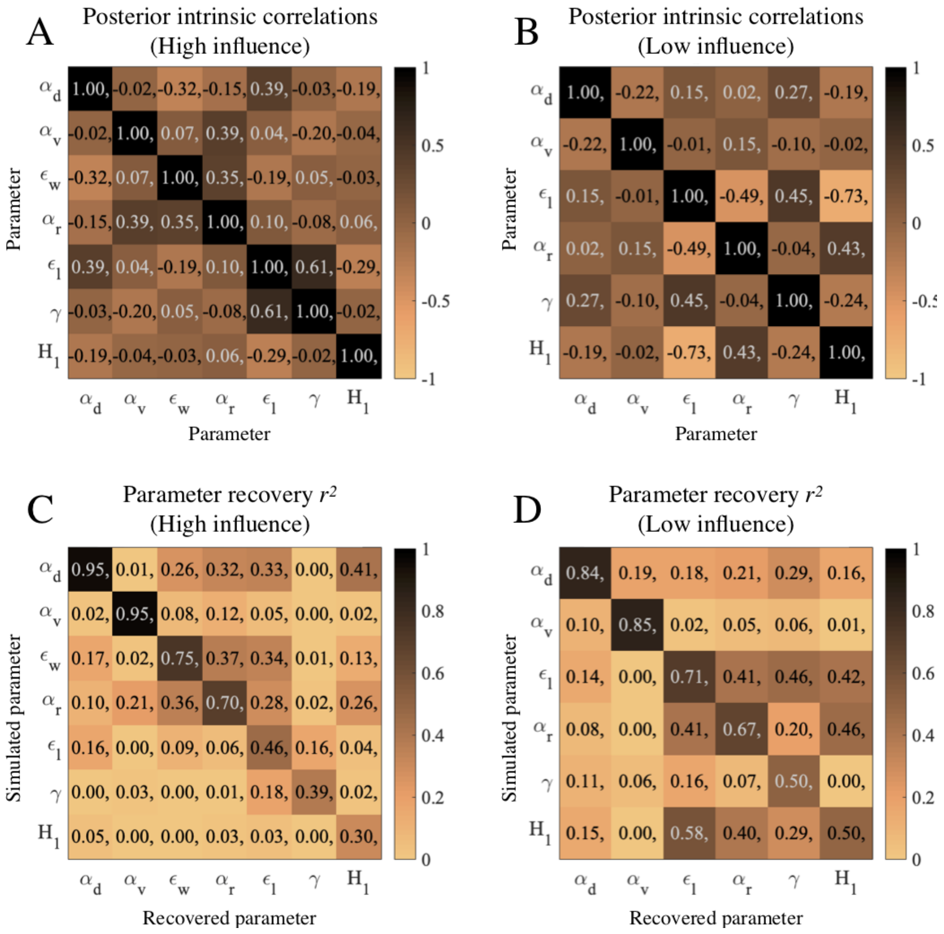

Figure 1: **Parameter recovery in DGTs. The confusion matrices exemplify the intrinsic correlations in the posterior parameter means (A; high influence and B; low influence conditions) and the capacity of our fitting procedure to recover the original parameters when these generate synthetic data (150 datasets; C, high influence; D, low influence conditions). For the latter two insets, we report the variance explained or r^2 as a clearer measure of how much information about the original parameters is carried by the recovered parameters. Recovery is quite good in high influence conditions, in which the variance explained for all parameters is maximal for the corresponding parameter (inset C). In particular with respect to our results and conclusions, recovery of the ϵ_l parameter is very good in high influence conditions. In low influence conditions, we find a measure of confusion between parameters H_1 and ϵ_l, which are respectively the initialisation, and the learning (from failure) components of the Rescorla-Wagner component of the model.**

(b) There may be something about the trial sequence that confounds vehicle-level learning with the optimal decision strategy. If this is plausible, the authors should validate this by fitting the ideal observer under different trial sequences.

We fit our ideal observer to the same double-goal trial sequences that participants were exposed to, which were in random order. Thus, our inferences about vehicle-level learning in the ideal observer cannot be due to it

being fit to specific sequences of trials.

Point 3. *We still believe that the authors are over-interpreting the implications of these results for the functional role of VMPFC. We suggest the reviewers limit their discussion of VMPFC to a discussion of future directions, for example suggesting that VMPFC may play a role in decision making in this task. The connection between controllability, aversive learning, and VMPFC is too speculative, given the nature of the data in this experiment. Even then, the RW model suggests that participants placed very little weight on controllability, and much more on reward, distance, and especially value-learning. To the extent that the implications for VMPFC depend on controllability, the authors should be cautious in generalizing the results from this experiment. Similarly, the authors should consider whether situating this paper within the controllability literature in their introduction does service to the cognitive strategies that appear to be employed in this task.*

We appreciate very much the point raised by the reviewer. We have further moderated our discussion of the possible implication of vmPFC, and, as requested by the reviewer, now simply make a suggestion as to a possible goal of a future study:

> [. . .] **Our study was not designed to reveal the neural substrates of the decision-making strategies involved, and in particular the excess learning from failure. From the original framing of the task as involving controllability, the vmPFC would be an obvious target of investigation, although one should note that, under our operationalization of this term, it had only a relatively modest impact on the behavior.** [. . .]

We only believe that vmPFC could be relevant because its activity has been shown to be moderated by LoC, and, while the reviewer is quite right that we have found only marginal implications of controllability (in terms of entropy) as we operationalised it, we still believe that the paper belongs to the literature on control, as it points to potential mechanisms of maintenance of a good (or poor) idea of one's own perceived control - making a bid to place a particular emphasis on aversive learning.
