# Supplementary material for: Internality and the internalisation of failure: Evidence from a novel task
Source: PLoS Comput Biol. 2021 Jul 6;17(7):e1009134. doi: 10.1371/journal.pcbi.1009134 (PMC8284820; doi:10.1371/journal.pcbi.1009134)
Supplement: Attachment — Submitted filename: ResponseToReviewers.pdf [file pcbi.1009134.s014.pdf]

Responses to reviewers

May 23, 2021

Dear reviewers,

We are very grateful for your efforts reviewing our manuscript, 'Internality and the Internalisation of Failure: Evidence From a Novel Task'. Please find our responses below, with original comments by each reviewer in cursive fonts, our response to each comment in normal fonts, and changes to the manuscript in bold fonts. When contents of tables, figures, or equations, are changed, we explicitly provide a reference to the corresponding element in text. All present changes have been added to the document using red font, so that these are identifiable.

Reviewer 1

We appreciated being part of this pilot, and thank the reviewers for their efforts in replicating our analyses.

Reviewer 2

The authors have diligently replied to our concerns, and we are satisfied (and impressed) with the contents of this paper, with some amendments to how the uncertainties raised by parameter recovery should be conveyed.

We thank the reviewer for the kind words and appreciate the point raised, which we address below.

We recommend softening the tone in interpreting the recovery of the learning-from-losses (ϵ_l) parameter: it shows strong posterior correlations with other parameters, and the recovery is on the low side, especially given the small sample size. More conservatively conveying your certainty in these analyses, in conjunction with the parameter recoveries you report in the supplement, will informative for a reader whose primary focus are these individual differences. Similarly, the caption for figure 6 should mention these difficulties in recovery.

We appreciate and agree with the reviewer's caution. We edited the main text and supplementary materials to make the issues in parameter recovery more explicit to the reader. Particularly, in main text (caption in Figure 6):

> **[...] however, note that the ϵ_l parameter in these conditions exhibits a measure of confusion, particularly with the initialisation (i.e. H_1) parameter(see Figure S5).**

We now also address the issue in Discussion:

[. . .] **From the perspective of determining individual differences, it would be desirable to increase the fidelity with which we can recover parameters (from simulated data). This might be achieved by presenting a single ('intermediate') influence condition, rather than two (since it was difficult, for instance, to disentangle low initialisation values from fast learning in low influence conditions). In this case, a single set of parameters would be constrained by all 160 trials.**[. . .]

Please also match the order of the parameters in FigS5 to FigS2, relabel the 'gamma' parameter in FigS5 to α_H*, and include the parameter names in the figure caption.*

As suggested, we rearranged the labels of figure S5. We also edited the caption of figure S5 to highlight the limitations in parameter recovery:

[. . .] **Here, we show recovery analyses results for the winning model parameters (i.e. sensitivities to distance,** α_d**; to reward,** α_r**; to guidability,** α_v**; learning gain,** α_H**; learning rates from success,** ϵ_w**; and from failure,** ϵ_l**; and the initialisation,** H_1**). The confusion matrices illustrate the intrinsic correlations in the posterior parameter means (A; high influence and B; low influence conditions) and the capacity of our fitting procedure to recover the original parameters when these generate synthetic data (150 datasets; C, high influence ;D, low influence conditions). For C;D, we report the variance explained or** r^2 **as a transparent measure of the amount of information about the original parameters that we can recover. Parameters** ϵ_l **and** α_H **parameters exhibit rather substantial correlations in the posterior (part A). However, recovery remains adequate (part C), and, in particular for our conclusions, of the** ϵ_l **parameter in high influence conditions. In low influence conditions, we find a measure of confusion between parameters** H_1 **and** ϵ_l**, which are respectively the initialisation, and the learning (from failure) components of the Rescorla-Wagner component of the model. Thus, functional interpretations of individual differences in this condition should be interpreted with caution.**[. . .]
